# Non-invasive brain stimulation for treating cognitive and neuropsychiatric non-motor symptoms in Parkinson’s disease and atypical parkinsonism: a systematic review and meta-analysis of randomized controlled trials

**DOI:** 10.1007/s00702-026-03127-x

**Published:** 2026-03-05

**Authors:** Elisa Mantovani, Eleonora Bertoncello, Mirko Filippetti, Alessandro Picelli, Michele Tinazzi, Stefano Tamburin

**Affiliations:** 1 Section of Neurology, Department of Neurosciences, Biomedicine and Movement Sciences, University of Verona, Piazzale L.A. Scuro 10, 37134 Verona, Italy; 2 Section of Physical Medicine and Rehabilitation, Department of Neurosciences, Biomedicine and Movement Sciences, University of Verona, Piazzale L.A. Scuro 10, 37134 Verona, Italy

**Keywords:** Parkinson’s disease, Atypical parkinsonism, Non-motor symptoms, Cognition, Neuropsychiatric manifestations, Non-invasive brain stimulation

## Abstract

Cognitive and neuropsychiatric non-motor symptoms (NMS) are common in Parkinson’s disease (PD) and atypical parkinsonism (AP), yet their management is difficult. Non-invasive brain stimulation (NIBS) may offer a therapeutic option. To provide a joint and comprehensive overview of studies on NIBS to treat cognitive and neuropsychiatric NMS in PD and AP. We searched for randomized, sham-controlled trials on repeated NIBS sessions for treating cognitive and neuropsychiatric NMS in PD and AP. Studies were categorised by NIBS technique/protocol, NMS, and stimulation target. We found 27 studies on repetitive transcranial stimulation (rTMS; N = 1309 patients, range 18–106) and 7 studies on transcranial direct current stimulation (tDCS; N = 221, 10–77). Cognition was assessed in 17 rTMS and 6 tDCS studies, depression by 24 rTMS and 4 tDCS studies, anxiety by 11 rTMS and 1 tDCS studies, apathy by 4 rTMS and 1 tDCS studies, psychosis by 1 tDCS study. Variability in NIBS targets and parameters, outcome measures, treatment duration and follow-up raised some concerns (N = 26) or high risk of bias (N = 8). The meta-analysis on PD suggested that, despite high heterogeneity and low-to-moderate certainty of evidence, excitatory rTMS of the left dorsolateral prefrontal cortex (DLPFC) and the primary motor cortex, and inhibitory rTMS of the right DLPFC may be effective for depression and anxiety. Methodological heterogeneity impeded robust conclusions on the efficacy of NIBS for cognitive and neuropsychiatric NMS in PD and AP. We propose a roadmap for the design of future studies.

**Supplementary Information:**

The online version contains supplementary material available at 10.1007/s00702-026-03127-x.

## Introduction

Cognitive and neuropsychiatric non-motor symptoms (NMS) are common and early manifestations of Parkinson's disease (PD) and atypical parkinsonism (AP), which includes Lewy body dementia (LBD), progressive supranuclear palsy (PSP), multiple system atrophy (MSA) and corticobasal degeneration (CBD) (Chaudhuri and Schapira 2009; Jellinger 2025).

Cognitive impairment in PD ranges from subjective cognitive decline (SCD), i.e., subjective complaints not affecting performance on objective cognitive tests, to mild cognitive impairment (MCI), i.e., mild disturbances with little impact on activities of daily living, and dementia, i.e., more widespread cognitive impairment and impact (Aarsland et al. 2021). AP is associated with more extensive patterns of executive, language and visuospatial cognitive impairment (Raimo et al. 2023).

Cognitive NMS of PD and AP have been associated to amyloid-β deposition, tau neurofibrillary tangles coexisting with α-synuclein leading to dysfunction of dopaminergic, serotonergic, noradrenergic, cholinergic, and glutamatergic system (Qamar et al. 2017; Mantovani et al. 2024b; Jellinger 2025).

Depression and anxiety are the most frequent neuropsychiatric NMS, affecting up to 30–40% of PD patients (Mantovani et al. 2023) and up to 60–70% of AP cases (Jellinger 2025). Dopaminergic, serotonergic, and noradrenergic changes in the limbic system and striatum contribute to depression in PD/AP (Galts et al. 2019), while anxiety is associated with noradrenergic and dopaminergic damage (Qamar et al. 2017).

Psychosis includes illusions, hallucinations, delusions, and paranoid ideation, with more severe manifestations occurring in parallel with cognitive decline and loss of insight (Ffytche et al. 2017; Mantovani et al. 2023). Visual hallucination, i.e., the most common PD-related psychosis, is ascribed to cholinergic damage and dysfunction of multiple brain pathways (Ignatavicius et al. 2025; Marinus et al. 2018). Psychosis is less common in AP (Jellinger 2025).

Apathy, i.e., a reduction or loss of motivation, occurs in 20–60% of PD patients (Pagonabarraga and Kulisevsky 2017) and up to 56% of PSP cases (Jellinger 2025). Apathy in PD is associated with dopamine, serotonin, and acetylcholine disruption in limbic circuits and the prefrontal and anterior cingulate cortex (ACC) (Morris et al. 2023; Pagonabarraga and Kulisevsky 2017; Qamar et al. 2017).

Impulse control disorders and related behaviors (ICDs) include addiction-like manifestations related to reward-based activities (i.e., pathological gambling, hypersexuality, binge eating, compulsive buying) and other repetitive, excessive, uncommon behaviors (i.e., hobbyism, punding, walkabout and dopamine dysregulation syndrome) with prevalence up to 35% in PD (Weintraub et al. 2022), and less frequently reported in PSP (Jellinger 2025). Dopamine replacement therapy plays a key role in ICDs by causing mesocorticolimbic dopaminergic overdose, but genetic predisposition and functional changes in the striatum, ACC, orbitofrontal cortex, cortico-striatal connectivity, and the default mode, salience, and central executive networks may contribute to their onset (Martini et al. 2020).

Anhedonia, impulsivity and akathisia are other neuropsychiatric NMS of PD and AP, whose underlying pathophysiology is unknown (Mantovani et al. 2023; Jellinger 2025).

Non-invasive brain stimulation (NIBS) includes repetitive transcranial magnetic stimulation (rTMS), transcranial direct current stimulation (tDCS), transcranial alternate current stimulation (tACS), transcranial random-noise stimulation (tRNS), low-intensity focused ultrasound (LIFU), and transcranial pulse stimulation (TPS) that may be applied with different protocols (Toth et al. 2024). High-frequency (HF, i.e., excitatory) rTMS of the left dorsolateral prefrontal cortex (DLPFC) has level A evidence of efficacy in depression and level B evidence in PD-related depression, low-frequency (LF, i.e., inhibitory) rTMS of the right DLPFC and bihemispheric stimulation of the DLPFC have level B evidence in depression (Lefaucheur et al. 2020), while anodal tDCS (i.e., excitatory) of the left DLPFC (with right orbitofrontal cathode) has level B evidence in major depressive episode without drug resistance (Lefaucheur et al. 2017). Anodal tDCS of the right DLPFC (with left DLPFC cathode) has level B evidence in addiction/craving (Lefaucheur et al. 2017), which is supposed to share pathophysiology with ICD in PD. LIFU can induce transient changes in neuronal excitability in deep brain areas, supposed to play a crucial role in cognitive/neuropsychiatric NMS. A very recent open-label study showed that LIFU is safe and potentially effective for reducing drug craving and use in substance use disorders (Rezai et al. 2025).

The aim of this manuscript is to collect evidence on NIBS for treating cognitive and neuropsychiatric NMS in PD and AP through a systematic review and to synthesize the results with a meta-analytical approach, providing a clearer separation of stimulation targets and effect on brain excitability. Finally, limitations of current studies and new approaches will be discussed to improve quality and robustness of evidence in future studies.

## Methods

This systematic review and meta-analysis was conducted following the recommendations provided by the Cochrane Handbook for Systematic Reviews of Interventions (Higgins et al. 2024) and the Preferred Reporting Items for Systematic and Meta-Analyses guidelines (Page et al. 2020). The research protocol was recorded in the International Prospective Register of Systematic Reviews PROSPERO (registration number: CRD42024500881) (Mantovani et al., 2024).

### Eligibility criteria

The PICOS framework was chosen to frame the inclusion criteria for this review. The *Population* (P) included adult (i.e., > 18 years) patients with an established diagnosis of PD or AP; the *Intervention* (I) of interest was any type of NIBS, including rTMS (i.e., HF, LF, deep, theta burst stimulation, TBS), tDCS, tACS, tRNS, TPS and FUS; the *Comparison* (C) was sham NIBS; the *Outcome* (O) consisted in group differences in the frequency/severity of cognitive and neuropsychiatric PD/AP-related NMS after intervention as primary/secondary outcome, comparing PD/AP patients undergoing active vs sham NIBS treatment; the *Study type* (S) encompassed randomized, sham-controlled trials evaluating the effects of repeated sessions of NIBS on PD/AP-related cognitive and neuropsychiatric NMS. Cross-over studies were only included if there was an adequate washout period (i.e., at least 4 weeks) before the cross-over. Trials combining NIBS and other pharmacological/non-pharmacological treatments were included only if a sham NIBS condition was provided as control. Open label trials, studies without a sham comparator, reports of single NIBS sessions, abstracts and conference proceedings, and studies with no therapeutic aim but only to assess neurophysiological measures were excluded.

### Information sources and search strategy

PubMed/MEDLINE, EMBASE and the Cochrane Library were searched from inception to November 10th, 2025 using a combination of terms related to PD, AP, NIBS and PD/AP-related cognitive and neuropsychiatric NMS (full search strings provided as supplementary material). Besides, the reference lists of relevant records and ClinicalTrials.gov were consulted for any additional citations potentially missed with the database search and unpublished data.

### Study selection

Search results were uploaded to Rayyan software, a web-based application that facilitates collaboration work among reviewers throughout the whole systematic review process (i.e., merging of records from multiple search engines, duplicates identification, titles and abstracts screening, full-texts screening, identification of conflicts between reviewers) (Ouzzani et al. 2016). Two authors (EM, ST) independently performed the study selection and disagreements were solved by consensus.

### Data extraction

A shared, previously pilot-tested data extraction form was created to record the following data: study design (i.e., parallel, cross-over), sample size, sex, age, disease duration, Hoehn and Yahr stage, Unified PD Rating Scale motor section (UPDRS-III), levodopa equivalent daily dose (LEDD), frequency/severity of cognitive (i.e., mild cognitive impairment, dementia) and neuropsychiatric features (i.e., anxiety, depression, apathy, ICD , other) at baseline, type of NIBS (e.g., rTMS, tDCS, other; NIBS only/combined with other treatments), NIBS protocol details (i.e., excitatory/inhibitory protocol, number of sessions/week, duration, washout period—in case of cross-over design—stimulation parameters, stimulation site, type of targeting, follow-up), outcome details (i.e., type, scale, primary/secondary), change in frequency/severity of cognitive and neuropsychiatric NMS after intervention/at follow-ups, nature/frequency of adverse events. Outcomes were presented as significant only if the significance was confirmed by between-groups statistical analysis.

### Risk of bias

Two independent reviewers (EM, ST) assessed the risk of bias by means of the Cochrane risk-of-bias tool for randomized trials (RoB 2.0) (Sterne et al. 2019). Any disagreement was solved by consensus. Each study was rated as carrying a “high” risk of bias, raising “some concerns” for risk of bias or having a “low” risk of bias, according to the following domains: bias arising from the randomization process; bias due to deviations from intended interventions; bias due to missing outcomes; bias in measurement of the outcomes; bias in selection of the reported results. A formal GRADE assessment was not performed, as providing recommendations on the clinical application of NIBS for the treatment of cognitive and neuropsychiatric NMS in PD and AP was beyond the scope of this paper.

### Data analysis

Data were synthesized according to a systematic and descriptive analysis of the results, which was provided in the text and tables to summarize the characteristics and findings of the included studies. For studies with crossover designs, we planned to analyze data from the first trial period only.

Separate meta-analyses were performed for cognitive and neuropsychiatric NMS, as well as for trials using transcranial magnetic and electrical NIBS, different brain targets, and different assessment timepoints (i.e., post-treatment, 1- or 3-month follow-up). Where studies reported more than one test/scale for a single cognitive/neuropsychiatric domain, recommended or more specific/sensitive measures were selected according to the indications provided by the International Parkinson and Movement Disorders Society (Schrag et al. 2007; Leentjens et al. 2008a, b; Skorvanek et al. 2018; Evans et al. 2019). The main outcomes of interest for the meta-analysis were post-treatment scores. Seven Authors were contacted regarding missing data; original datasets were obtained from the corresponding Author of one study (Trung et al. 2019).

Data were analyzed using Review Manager (The Cochrane Collaboration 2020). Mean differences (MD) and standardized mean differences (SMD) were chosen as effect size measures since the outcomes of interest were either reported using the same or different tests/scales and were calculated from reported means and standard deviations of cognitive and neuropsychiatric outcomes, with 95% confidence intervals (CI). Heterogeneity between studies was explored using the heterogeneity statistic (I^2^), with I^2^ of 0–25%, 26–50%, 51–75%, 76–100% indicating low, moderate, substantial and considerable heterogeneity, respectively. As the included studies were quite heterogeneous in terms of population and outcome measures, random-effect models were applied. Publication bias was planned to be assessed by inspecting funnel plots. Sensitivity and moderator analyses were planned to be conducted depending on the results of heterogeneity and the number of included studies per outcome, respectively (Borenstein et al. 2009). The level of statistical significance was set at 5% and 95% CIs were calculated. The results were presented graphically using forest plots.

## Results

### Identification and selection of the studies

**Fig. 1:**
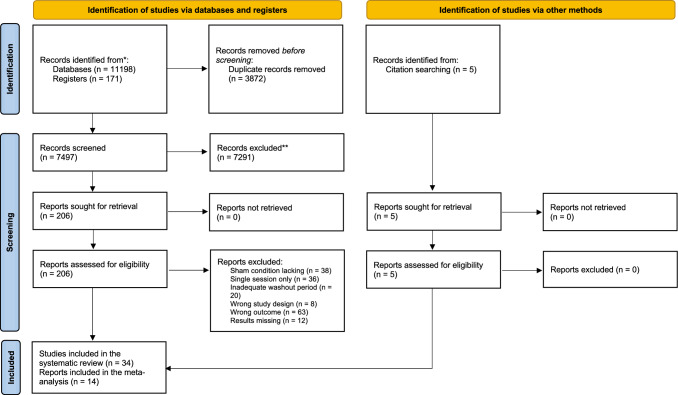
PRISMA diagram of the study. Page et al. (2021); www.prisma-statement.org

A total of 11,369 records were identified through literature search. After duplicates removal, 7497 records were screened through titles and abstracts, and 206 reports were obtained for full-text screening. Two authors (EM, ST) independently evaluated the 206 selected reports for in-depth examination. Disagreement concerned three reports (inter-rater agreement: 97%) and was solved by discussion. Thirty-four reports fulfilled the inclusion criteria and were therefore included in the systematic review; fourteen (Makkos et al. 2016; Shin et al. 2016; Brys et al. 2016; Trung et al. 2019; Khedr et al. 2020; Zhuang et al. 2020; Lang et al. 2020; He et al. 2021; Wei et al. 2022; Jiang et al. 2023; Song et al. 2024; Wu et al. 2024; Zhang et al. 2025; Feng et al. 2025) of them were also included in the meta-analysis (Fig. 1).

Studies were grouped according to the NIBS technique used (i.e., rTMS: N = 27; tDCS: N = 7) and for the rTMS protocol (i.e., excitatory: N = 18; inhibitory: N = 6; different protocols: N = 3). No studies on tACS, tRNS, LIFU or tPS were found.

### Studies on rTMS protocols

#### Types of rTMS protocols, targets and PD/AP populations

Twenty-seven reports explored rTMS protocols for cognitive and neuropsychiatric NMS (Okabe et al. 2003; Hamada et al. 2008; Arias et al. 2010; Pal et al. 2010; Benninger et al. 2012; NCT00955032 ReStore 2012; Shirota et al. 2013; Makkos et al. 2016; Shin et al. 2016; Brys et al. 2016; Buard et al. 2018; Cohen et al. 2018; Trung et al. 2019; Khedr et al. 2020, 2024; Zhuang et al. 2020; Li et al. 2020; Lang et al. 2020; He et al. 2021; Pan et al. 2022; Wei et al. 2022; Jiang et al. 2023; Song et al. 2024; Barboza et al. 2024; Wu et al. 2024; Zhang et al. 2025; Feng et al. 2025) (Table 1). All of them included patients with PD, except one on MSA (Pan et al. 2022). Twenty studies used excitatory rTMS protocols (HF, N = 15; intermittent TBS, iTBS, N = 3) to target the left (N = 8), left mid (N = 1) or bilateral (N = 1) DLPFC (Pal et al. 2010; Shin et al. 2016; Trung et al. 2019; Lang et al. 2020; He et al. 2021; Pan et al. 2022; Wei et al. 2022; Jiang et al. 2023), bilateral (N = 4) or unilateral M1 (N = 1) (Benninger et al. 2012; Makkos et al. 2016; Khedr et al. 2020; Li et al. 2020; Song et al. 2024), bilateral SMA (N = 1) (Hamada et al. 2008), bilateral parietal areas (N = 1) (Khedr et al. 2024), right posterior insula (N = 1) (Barboza et al. 2024), while two studies compared left DLPFC, bilateral M1 and their combined stimulation (Brys et al. 2016) and bilateral M1, SMA and their combined stimulation (Zhang et al. 2025). Six studies used inhibitory LF rTMS of the right DLPFC (N = 3) (Zhuang et al. 2020; Wu et al. 2024; Feng et al. 2025), bilateral M1 (N = 2) (Okabe et al. 2003; Arias et al. 2010), M1 and the prefrontal cortex (PFC, N = 1) (Buard et al. 2018). A single study compared HF and LF rTMS of the SMA (Shirota et al. 2013). There was a high heterogeneity of rTMS sessions across studies (i.e., 5–24). Tables S1 and S2 report clinical data of the PD and AP populations.

**Table 1:** Studies on TMS treatment for cognitive and neuropsychiatric NMS in PD and AP patients

References	Study design	Sample size^a^	Stimulation site and coil	TMS protocol details	Outcomes^b^	Timepoints	Follow-up	Adverse events	Results^c^
*Studies on HF rTMS (excitatory protocol)—PD*									
Hamada et al. (2008)	RCT, multicenter, parallel design, sham-controlled	93/99	SMA (figure-of-8 coil)	1 session/week over 8 weeks, 5 Hz, 1000 pulses/session, 110% AMT, targeting by anatomical landmarks	Depression (HAM-D, secondary)	Baseline, end of trt, 2.5, 3 mos after trt	2.5, 3 mos after trt	NR	No significant difference between real and sham stimulation
Pal et al. (2010)	RCT, parallel design, sham-controlled	22/22	Left DLPFC (figure-of-8 coil)	1 session/day over 10 days, 5 Hz, 600 pulses/session, 90% RMT, targeting by anatomical landmarks	Depression (MADRS, BDI; primary), cognition (MMSE, TMT-A, B, Stroop test-accuracy, secondary)	Baseline, end of trt, 1 mo after trt	1 mo after trt	Headache (real, N = 2)	Significant difference between real and sham stimulation for MADRS, BDI and Stroop test-accuracy scores up to 1 mo
Benninger et al. (2012)	RCT, parallel design, sham-controlled	26/26	M1 (circular coil, both sides in sequence)	8 sessions over 2 weeks, 50 Hz, 80% AMT, targeting by hand muscle MEP	Depression (BDI), cognition (FAB), secondary	Baseline, end of trt, 1 month after trt	1 mo after trt	None	No significant difference between real and sham stimulation
NCT00955032 (ReStore, 2012)	RCT, parallel design, sham-controlled	24/24	Left mid-DLPFC (figure-of-8 coil)	10 sessions, 5 Hz, 2000 pulses/session, 90% RMT, targeting not specified	Apathy (AES, primary; LARS, secondary), depression (BDI-II, HAM-D, secondary)	Baseline, end of trt	None	Headache (real, N = 2), fatigue (real/sham, N = 1), hallucination (sham, N = 1), back pain (real/sham, N = 1), PD worsening (real, N = 3), fall (real, N = 2), edema (real, N = 2)	Results presented as mean (SD) with no statistical analysis; no significant difference between real and sham stimulation (unpaired t-test)
Shin et al. (2016)	RCT, parallel design, sham-controlled	18/21	Left DLPFC (figure-of-8 coil)	5 sessions/week over 2 weeks, 5 Hz, 600 pulses/session, 90% RMT, targeting by anatomical landmarks	Depression (HAM-D, MADRS, BDI, primary)	Baseline, end of trt, 1 mo after trt	1 mo after trt	Headache (real, N = 1), facial muscle contraction (sham, N = 1)	Significant difference between real and sham stimulation for HAM-D and MADRS scores up to 1 mo
Makkos et al. (2016)	RCT, parallel design, sham-controlled	44/46	M1 (circular coil, both sides in sequence)	1 session/day over 10 consecutive days, 5 Hz, 600 pulses/session, 90% RMT, targeting by hand muscle MEP	Depression (BDI, MADRS, primary), cognition (MMSE, MoCA, Stroop, TMT, secondary)	Baseline, end of trt, 1 mo after trt	1 mo after trt	None	Significant difference between real and sham stimulation for BDI and MADRS up to 1 mo
Buard et al. (2018)	RCT, parallel design, sham-controlled	46/46	DLPFC (figure-of-8 coil, both sides in sequence)	1 session/day over 10 consecutive days, 20 Hz, 1500 pulses/session, 90% RMT, targeting by neuro-navigation based on standard MRI	Cognition (DRS-2, primary; TMT-B, DKEFS verbal/letter fluency, DKEFS color-word interference task, SDMT, CVLT-II, BNT, BTA, JLO), anxiety, depression (HADS), secondary	Baseline, end of trt	None	Headache (real, N = 1), transient faint (real, N = 1), blurry vision (real, N = 1)	No significant difference between real and sham stimulation
Khedr et al. (2020)	RCT, parallel design, sham-controlled	33/36	M1 (figure-of-8 coil, both sides in sequence)	5 sessions/week for 2 weeks, 20 Hz, 2000 pulses/session, 90% RMT, 5 booster sessions/mo for 3 mos, targeting by hand muscle MEP	Cognition (MMSE, MoCA, MES, CDR), primary/secondary not specified	Baseline, end of trt, 1, 2, 3 mos after trt	1, 2, 3 mos after trt	NR	Significant difference between real and sham stimulation for MoCA and MMSE after trt
Li et al. (2020)	RCT, parallel design, sham-controlled	48/48	M1 (figure-of-8 coil, left/right depending on pain distribution)	1 session/day for 5 consecutive days, 20 Hz, 2000 pulses/session, 80% RMT, targeting by hand muscle MEP	Depression (HAM-D), anxiety (HAM-A), secondary	Baseline, end of trt, 2 weeks, 1 mo after trt	2 weeks, 1 mo after trt	Headache (real, N = 3), tinnitus (real, N = 2), gait worsening (real, N = 1)	Significant difference between real and sham stimulation for HAM-D and HAM-A up to 1 mo
Wei et al. (2022)	RCT, parallel design, sham-controlled	60/60	Left DLPFC (figure-of-8 coil)	2 sessions/weekday for 2 weeks, 5 Hz, 1200 pulses/session, 110% RMT, targeting by neuro-navigation based on single subject MRI	Cognition (ANT, primary; MoCA, TMT-A, B, WCST, Stroop test-accuracy, RT and interference, secondary)	Baseline, end of trt,	None	NR	Significant difference between real and sham stimulation for WCST (categories completed), Stroop (RT and interference) and ANT (RT)
Jiang et al. (2023)	RCT, parallel design, sham-controlled	55/60	Left DLPFC (figure-of-8 coil)	1 session/day over 10 consecutive days, 10 Hz, 1200 pulses/stimulation, 100% RMT, targeting by neuro-navigation based on single subject MRI	Depression (HAM-D, primary), anxiety (HAM-A), cognition (MMSE, MoCA), secondary	Baseline, end of trt, 2 weeks, 1 mo after trt	2 weeks, 1 mo after trt	Headache (real, N = 2)	Significant difference between real and sham stimulation for HAM-D up to 1 mo and HAM-A only at follow-up
Khedr et al. (2024)	RCT, parallel design, sham-controlled	24/24	Parietal areas (figure-of-8 coil, both sides in sequence)	1 session/weekday over 2 weeks, 20 Hz, 2000 pulses/side, 80% RMT, targeting by anatomical landmarks	Depression (BDI-II, secondary)	Baseline, 10 days after trt	10 days after trt	NR	Significant difference between real and sham stimulation for real TMS on BDI-II
Barboza et al. (2024)	RCT, parallel design, sham-controlled	25/27	Right posterior-superior insula (double cone coil)	1 session/weekday over 1 week + 1 session/week over 7 weeks, 10 Hz, 3000 pulses/session, 80% RMT, targeting by neuro-navigation based on single subject MRI	Depression (HADS-D), anxiety (HADS-A), cognition (MMSE), secondary	Baseline, end of trt	None	Headache (real, N = 2)	No significant difference between real and sham stimulation
Song et al. (2024)	RCT, parallel design, sham-controlled	44/48	M1 (figure-of-8 coil, both sides in sequence)	1 session/day over 10 days, 10 Hz, 1000 pulses/session, 90% RMT, targeting by hand muscle MEP	Depression, (HAM-D), anxiety (HAM-A), cognition (MMSE, MoCA), secondary	Baseline, end of trt, 1 mo after trt	1 mo after trt	Dizziness/headache (real, N = 2), scalp numbness (sham, N = 1)	Significant difference between real and sham stimulation for HAM-D and HAM-A up to 1 mo
*Studies on HF rTMS (excitatory protocol)—AP*									
Pan et al. (2022)^d^	RCT, parallel design, sham-controlled	22/22	Left DLPFC	1 session/weekday for 2 weeks, 10 Hz, 1200 pulses/session, 100% RMT, targeting not reported	Depression (HAM-D), anxiety (HAM-A), secondary	Baseline, end of trt, 2 weeks, 1 mo after trt	2 weeks, 1 mo after trt	Headache (real, N = 2), dizziness (sham, N = 1)	Significant time x group interaction for HAM-D and HAM-A but no between group comparison reported
*Studies on iTBS (excitatory protocol)*									
Trung et al. (2019)	RCT, parallel design, sham-controlled	28/28	Left DLPFC (figure-of-8 coil)	2 session/day over 3 days (with 1–2 days in between), TBS: 3 pulses, 50 Hz, ISI 200 ms every 10 s, 600 pulses/session, 80% AMT, targeting by neuro-navigation based on single subject MRI	Cognition (overall, cognitive domains, primary), depression (BDI), anxiety (BAI), apathy (AES), secondary	Baseline, end of trt, 10 days, 1 mo after trt	10 days, 1 mo after trt	NR	No significant difference between real and sham stimulation
Lang et al. (2020)	RCT, parallel design, sham-controlled	41/43	Left DLPFC (figure-of-8 coil)	6 sessions over 1 week, TBS: 3 pulses, 50 Hz, ISI 200 ms every 10 s, 600 pulses/session, 80% AMT, targeting by neuro-navigation based on single subject MRI	Cognition (cognitive domains, primary), depression (BDI), anxiety (BAI), secondary	Baseline, 1 day, 1 mo after trt	1 mo after trt	Uncomfortable sensation in the face, (group/N NR)	No significant difference between real and sham stimulation
He et al. (2021)	RCT, parallel design, sham-controlled	35/40	Left DLPFC	1 session/weekday over 2 weeks, TBS: 3 pulses, 50 Hz, ISI 200 ms every 10 s, 600 pulses/session, 100% RMT, targeting by anatomical landmarks	Cognition (MoCA, RBANS, primary), depression (BDI, secondary)	Baseline, end of trt, 3 mos after trt	3 mos after trt	NR	Significant difference between real and sham stimulation for MoCA and RBANS up to 3 mos
*Studies on LF rTMS (inhibitory protocol)*									
Okabe et al. (2003)	RCT, parallel design (three arms), sham-and occipital stimulation-controlled	85/85	M1 (Cz, circular coil); occipital cortex (inion, circular coil)	Motor/occipital cortex stimulation: 1 session/week over 8 weeks, 0.2 Hz, 100 pulses/session, 110% AMT, targeting by anatomical landmarks	Depression (HAM-D, secondary)	Baseline, 1, 2 (end of trt), 3, 4 mos after trt	3, 4 mos after trt	NR	No significant difference between motor, occipital and sham stimulation
Arias et al. (2010)	RCT, parallel design, sham-controlled	18/18	M1 (Cz, circular coil)	5 sessions/week over 2 weeks, 1 Hz, 100 pulses/session, 90% RMT, targeting by anatomical landmarks	Depression (HAM-D, secondary)	Baseline, end of trt, 1 week after trt	1 week after trt	NR	No significant difference between real and sham stimulation
Cohen et al. (2018)	RCT, parallel design, sham-controlled	42/48	M1 + PFC (both sites in sequence, H5 coil)	3 sessions/week, 2 sessions/week and 1 session/week over 3 months, 1 Hz, 900 pulses/session, 110% MT (M1) + 10 Hz, 800 pulses/session, 100% MT (PFC), targeting by hand muscle MEP for M1 and by anatomical landmarks for left PFC	Depression (BDI), cognition (DSBT, DSFT, phonemic and semantic fluencies), secondary	Baseline, end of trt	None	Headache (real, N = 8; sham, N = 2), dizziness (real, N = 4), nausea, weakness, gait worsening (real/sham, N = 1),	No significant difference between real and sham stimulation
Zhuang et al. (2020)	RCT, parallel design, sham-controlled	33/33	Right DLPFC (figure-of-8 coil)	1 session/day over 10 days, 20 min/session, 1 Hz, 1200 pulses/session, 110% RMT, targeting by anatomical landmarks	Depression (HAM-D), cognition (MoCA), secondary	Baseline, end of trt, 1, 3, 6 mos after trt	1, 3, 6 mos after trt	Headache (real, N = 2)	Significant difference between real and sham stimulation for HAM-D (up to 3 mos) and MOCA (up to 6 mos)
Wu et al. (2024)	RCT, parallel design, sham-controlled	63/74	Right DLPFC (figure-of-8 coil)	1 session/day over 10 days, 1 Hz, 1200 pulses/session, 80% RMT, targeting by anatomical landmarks	Cognition (MoCA, secondary)	Baseline, end of trt, 3 mos after trt	3 mos after trt	NR	Significant difference between real and sham stimulation for MoCA scores (up to 3 mos)
Feng et al. (2025)	RCT, parallel design, sham-controlled	80/80	Right DLPFC	1 session/day over 10 days, 20 min/session, 1 Hz, 90% RMT, targeting by anatomical landmarks	Depression (HAM-D), anxiety (HAM-A), secondary	Baseline, 1 mo after trt	1 mo after trt	NR	Significant difference between real and sham stimulation for HAM-D and HAM-A (up to 1 mo)
*Studies comparing different TMS protocols*									
Shirota et al. (2013)	RCT, multicenter, parallel design (three arms), sham-controlled	102/106	SMA (figure-of-8 coil at midline)	1 session/week over 8 weeks, real LF (1 Hz), real HF (10 Hz), sham (10 Hz), 1000 pulses/session, 110% AMT, targeting by anatomical landmarks	Depression (HAM-D), apathy (apathy score), secondary	Baseline, end of trt, 2.5, 4, 5 mos after trt	2.5, 4, 5 mos after trt	None	No significant difference between real LF, real HF and sham stimulation
Brys et al. (2016)	RCT, parallel design (four arms), sham-controlled	50/61	M1 (both sides in sequence), left DLPFC, combined M1 + left DLPFC	5 sessions/week over 2 weeks, 10 Hz, 1000 pulses/session for each M1, 2000 pulses/session for left DLPFC, targeting by hand muscle MEP for M1 and by anatomical landmarks for left DLPFC	Depression (HAM-D, primary; BDI-II, secondary), anxiety (CAS), apathy (AES), cognition (MoCA), secondary	Baseline, end of trt, 1, 3 and 6 mos after trt	1, 3 and 6 mos after trt	Headache (real, N = 25)	No significant difference between real and sham stimulation and between combined vs. single site TMS
Zhang et al. (2025)	RCT, parallel design (four arms), sham-controlled	79/84	M1, SMA, combined M1 + SMA (both sides in sequence, figure-of-8 coil)	1 session/day over 10 days, 10 Hz, 1200 pulses/session, 100% RMT, targeting by hand muscle MEP and anatomical landmarks	Depression (HAM-D), anxiety (HAM-A), cognition (MMSE, MoCA), secondary	Baseline, end of trt, 1 mo after trt	1 mo after trt	Headache (N = 2), dizziness with nausea (N = 2), tinnitus (N = 1)	Significant time x group interaction for HAM-D and HAM-A but no between group comparison reported

Studies are reported in chronological order of publication

*AES* Apathy Evaluation Scale, *AMT* active motor threshold, *ANT* Attentional Network Test, *AP* atypical parkinsonism, *AS* Apathy Scale, *BDI* Beck Depression Inventory, *BNT* Boston Naming Test, *BTA* Brief Test of Attention, *CDR* Clinical Dementia Rating Scale, *CVLT-II* California Verbal Learning Test-version II, *DKEFS* Delis-Kaplan Executive Function System, *DLPFC* dorsolateral prefrontal cortex, *DRS-2* Dementia Rating Scale-2, *DSBT/DSFT* digit span backward/forward test, *dTMS* deep transcranial magnetic stimulation, *HADS* Hospital Anxiety and Depression Scale, *HAM-A* Hamilton Anxiety Scale, *HAM-D* Hamilton Depression Scale, *HF* high frequency, *iTBS* intermittent theta burst stimulation, *JLO* Judgement of Line Orientation task, *LF* low frequency, *M1* primary motor cortex, *MADRS* Montgomery-Asberg Depression Rating Scale, *MEP* motor evoked potential, *MES* Memory and Executive screening Scale, *MMSE* Mini Mental State Examination, *mo/mos* month/months, *MoCA* Montreal Cognitive Assessment, *M1* primary motor cortex, *MRI* magnetic resonance imaging, *MSA* multiple system atrophy, *NMS* non-motor symptoms, *NMSQ* Non-Motor Symptom Questionnaire, *NMSS* Non-Motor Symptoms Scale, *NR* not reported, *PD* Parkinson’s disease, *PFC* prefrontal cortex, *RBANS* Repeatable Battery for the Assessment of Neuropsychological Status, *RCT* randomized controlled trial, *RMT* resting motor threshold, *RT* reaction time, *rTMS* repetitive transcranial magnetic stimulation, *SD* standard deviation, *SDMT* Symbol Digit Modalities Test, *SMA* supplementary motor area, *TMT* Trail Making Test, *trt* treatment, *WAIS-R* Wechsler Adult Intelligence Scale-Revised, *WCST* Wisconsin Card Sorting Test

^a^Number of patients who completed the study and were analysed/number of included patients

^b^Type of outcome (scale), primary/secondary outcome of the study

^c^Significance is reported for between group (real vs. sham or different TMS groups) analyses

^d^Study including patients with MSA

#### Cognitive NMS

Cognitive outcomes were explored in 17 studies (primary, N = 3; secondary, N = 12; not specified, N = 2). Measures of global cognition (e.g., MoCA/MMSE) improved to real vs sham rTMS in 3 out of 12 studies (left DLPFC HF, N = 0/3 Pal et al. 2010; Wei et al. 2022; Jiang et al. 2023); bilateral M1 HF, N = 1/3 (Makkos et al. 2016; Khedr et al. 2020; Song et al. 2024); right posterior insula, N = 0/1 (Barboza et al. 2024); left DLPFC, bilateral M1, combined stimulation, N = 0/1 (Brys et al. 2016); left DLPFC iTBS, N = 1/2 (Trung et al. 2019; He et al. 2021); right DLPFC LF, N = 2/2 (Zhuang et al. 2020; Wu et al. 2024), with effects after right DLPFC LF lasting up to 3–6 mos. Several cognitive domains were explored as secondary outcomes in 10 studies (left/bilateral DLPFC HF rTMS, N = 4; Pal et al. 2010; Buard et al. 2018; He et al. 2021; Wei et al. 2022; bilateral M1 HF rTMS, N = 3; Benninger et al. 2012; Makkos et al. 2016; Khedr et al. 2020; left DLPFC iTBS, N = 2; Trung et al. 2019; Lang et al. 2020; combined M1 and PFC LF rTMS, N = 1; Cohen et al. 2018), with attention and executive function domains (Stroop test, Wisconsin card sorting test, attention network test) being improved after real vs sham treatment in 2 studies (Pal et al. 2010; Wei et al. 2022) and the effect on Stroop test lasting up to 1 month follow-up in a single study (Pal et al. 2010).

#### Neuropsychiatric NMS

Depression was the most common NMS, being explored as a primary/secondary outcome in 24 studies. Three out of 6 RCTs found significant improvement in measures of depression up to 1 month follow-up after real vs sham HF rTMS of the left DLPFC (Pal et al. 2010; Shin et al. 2016; Jiang et al. 2023). Three out of 4 studies reported real vs sham HF fTMS over bilateral (N = 2) (Makkos et al. 2016; Song et al. 2024) or unilateral M1 (N = 1) (Li et al. 2020) to be effective on depression up to 1 month after the treatment. Real HF rTMS on bilateral parietal areas was found to improve depression vs sham with no follow-up data (Khedr et al. 2024). Two out of 4 studies found improvement in depression up to 3 months follow-up after real vs sham LF rTMS of the right DLPFC (Zhuang et al. 2020; Feng et al. 2025). Three studies on iTBS of the left DLPFC (Trung et al. 2019; Lang et al. 2020; He et al. 2021) and four studies on HF rTMS targeting the SMA (N = 2) (Hamada et al. 2008; Shirota et al. 2013), posterior insula (N = 1) (Barboza et al. 2024) and M1, left DLPFC or their combination (N = 1) (Brys et al. 2016) yielded negative findings on depression.

Anxiety was the secondary outcome in 11 studies (Brys et al. 2016; Buard et al. 2018; Trung et al. 2019; Li et al. 2020; Lang et al. 2020; Pan et al. 2022; Jiang et al. 2023; Song et al. 2024; Barboza et al. 2024; Zhang et al. 2025; Feng et al. 2025) exploring inhibitory and excitatory rTMS. Although baseline anxiety severity was unbalanced, LF rTMS targeting the right DLPFC was found to improve anxiety at 1 month follow-up (Feng et al. 2025). The remaining studies (HF: bilateral/unilateral M1, N = 2/2; left DLPFC, N = 2/3; right posterior insula, N = 0/1; left DLPFC, bilateral M1, combined stimulation, N = 0/1; left DLPFC iTBS, N = 0/2; bilateral M1, SMA, combined stimulation, N = 1) showed real vs. sham HF rTMS to improve anxiety up to 1 month after treatment (Li et al. 2020; Pan et al. 2022; Jiang et al. 2023; Song et al. 2024; Zhang et al. 2025).

Apathy was the secondary outcome in 4 studies (left DLPFC HF rTMS, N = 2; Trung et al. 2019; left DLPFC, bilateral M1, combined HF rTMS, N = 1; Brys et al. 2016; SMA HF/LF rTMS, N = 1; Shirota et al. 2013) that yielded negative findings.

### Studies on tDCS protocols

#### Types of tDCS protocols, targets and PD/AP populations

Seven studies explored tDCS protocols for cognitive and neuropsychiatric NMS (Benninger et al. 2010; Doruk et al. 2014; Elder et al. 2019; Manor et al. 2021; Aksu et al. 2022; Simonetta et al. 2024; Cappiello et al. 2024) (Table 2). Two studies included patients with AP (i.e., LBD, PSP) (Elder et al. 2019; Cappiello et al. 2024). Anodal tDCS targeted the left/right DLPFC with supraorbital cathode (Doruk et al. 2014), the left DLPFC with the cathode on the right DLPFC (Aksu et al. 2022) or the left DLPFC with the cathode over the right deltoid muscle (Cappiello et al. 2024), the left M1 with supraorbital cathode (Simonetta et al. 2024), the left M1 and DLPFC with four contralateral/ipsilateral cathodes (Manor et al. 2021), bilateral M1 and PFC with mastoid cathode (Benninger et al. 2010) and the right posterior parietal cortex with the cathode over the occipital cortex (Elder et al. 2019). There was a high heterogeneity in the number of tDCS sessions across studies (i.e., 8–15). Tables S1 and S2 report clinical data of the PD and AP populations.

**Table 2:** Studies on tDCS treatment for PD and AP-related cognitive and neuropsychiatric NMS

References	Study design	Sample size^a^	Stimulation site/montage	Protocol details	Outcome measures^b^	Timepoints analysis	Follow-up	Adverse events	Results^c^
*Studies on PD*									
Benninger et al. (2010)	RCT, parallel design, sham-controlled	25/25	Real: M1/PFC anode (consecutive stimulation, 4 times/area), mastoid cathode; sham: forehead anode/cathode, 2 inactive mastoid electrodes	8 sessions over 2.5 weeks; real: 2 mA for 20 min; sham: 1 mA for 1–2 min	Depression (BDI), secondary	Baseline, end of trt, 1, 3 mos after trt	1, 3 mos after trt	Scalp burn (N = 1), tingling (all patients)	No significant difference between real and sham stimulation
Doruk et al. (2014)	RCT, multicenter, parallel design (three arms), sham-controlled	18/18	Real: left DLPFC (F3) anode, right supraorbital region cathode; right DLPFC (F4) anode, left supraorbital region cathode; sham: random left/right DLPFC anode, contralateral supraorbital region cathode	1 session/weekday over 2 weeks; real: 2 mA for 20 min; sham: 2 mA for the initial 30 s ramp up/down	Cognition (MMSE, TMT-A, -B, WCST, PCL, WM, Stroop test, HPVOT, CPM, DSFT, DSBT, 3-Back Test), depression (BDI, HRSD, anxiety (HAS), primary/secondary not specified	Baseline, end of trt,1 mo after trt	1 mo after trt	Tingling (N = 9), sleepiness (N = 10), headache/neck pain (N = 12), skin redness (N = 4), trouble concentrating (N = 4)	No significant difference between real and sham stimulation at the end of trt, significant improvement in TMT-B after 1 mo for both real tDCS groups vs. sham
Manor et al. (2021)	RCT, parallel design, sham-controlled	45/77	Real: Left DLPFC + M1 (lower limbs), F3, Cz anode, AF4, FC1, FC5, CP1 cathode; sham: Cz, FC1 anode, F3, CP1 cathode	1 session/weekday over 2 weeks (intensive) + 1 session/week over 5 weeks (maintenance); real: 4 mA for 20 min; sham: immediate ramp down to 0	Cognition (Neurotrax EF score), secondary	Baseline, end of intensive trt, 10 weeks after maintenance	10 weeks after maintenance	Headache/neck pain (real: N = 4), scalp burn (real: N = 4), skin redness (real: N = 5), sleepiness (real: N = 12, trouble concentrating (real: N = 2), mood change (real: N = 3)	No significant difference between real and sham stimulation
Aksu et al. (2022)	RCT, parallel design, sham-controlled	26/26	Bilateral DLPFC, L-DLPFC (F3) anode, R-DLPFC (F4) cathode	2 sessions/day over 5 days; real: 2 mA for 20 min; sham: immediate ramp down to 0	Cognition (TMT A, DSFT, Stroop test, COWAT, BJLO, Benton’s facial recognition test, Oktem Verbal Memory Processes Test, WMS R – Logical Memory, BNT, semantic fluency, CPT), primary/secondary not specified	Baseline, end of trt, 1 mo after trt	1 mo after trt	NR	No significant difference between real and sham stimulation
Simonetta et al. (2024)	RCT, crossover design (3- mo whashout)	10/10	Left M1 (C3) anode, right supraorbital ridge (Fp2) cathode	1 session/weekday over 2 weeks; real: 2.0 mA for 20 min; sham: ramp down to 0 after 40 s	Cognition (PD-CRS), secondary	Baseline, end of trt	None	None	No significant difference between real and sham stimulation
*Studies on AP*									
Elder et al. (2019)^d^	RCT, parallel design, sham-controlled	29/40	Right posterior parietal cortex (P4) anode, Oz cathode	2 sessions/weekday over 4 days; real: 7 s fade-in then 0.048 mA/cm^2^ for 20 min; sham: 7 s fade-in then stop	Hallucinations (NPI), primary; cognition (MMSE, CAMCOG, TMT-A, -B, phonemic fluency, computerized attentional and visuoperceptual tasks), depression (GDS-15), secondary	Baseline, end of trt, 1, 3 mos after trt	1, 3 mos after trt	None	No significant difference between real and sham stimulation
Cappiello et al. (2024)^e^	RCT, parallel design, sham-controlled	25/25	Left DLPFC (F3) anode, right deltoid muscle cathode	1 session/weekday over 2 weeks; real: 2 mA for 20 min; sham: ramp down to 0 after 5 s	Cognition (verbal and semantic fluencies, primary; MoCA, FAB, secondary); neuropsychiatric disturbances (NPI), depression (BDI-II), apathy (AES), secondary	Baseline, end of trt, 45 days and 3 mos after trt	45 days and 3 mos after trt	Headache/neck/skin pain (real: 7, sham: 6), tingling/burning sensation (real: 14, sham: 11), skin itching/reddening (real: 15, sham: 7), drowsiness (real: 7, sham: 6), difficulty in concentrating (real: 6, sham: 7), psychiatric events (real: 1, sham: 7), perception of stimulation (real: 12, sham: 7), falls (real: 3, sham: 1), other (real: 1, sham: 1),	No significant difference between real and sham stimulation

Studies are reported in chronological order of publication

*AES* Apathy Evaluation Scale, *APD* atypical parkinsonisms, *BDI* Beck Depression Inventory, *BJLO* Benton’s judgement of line orientation test, *BNT* Boston Naming Test, *CAMCOG* Cambridge Cognitive Examination, *CPM* Colored Progressive Matrices, *COWAT* Controlled Word Association Test, *CPT* Continuous Performance Test, *DLPFC* dorsolateral prefrontal cortex, *DSBT* digit span backward test, *DSFT* digit span forward test, *EF* executive function, *FAB* Frontal Assessment Battery, *HAS* Hamilton Anxiety Scale, *HRSD* Hamilton Depression Rating Scale, *HPVOT* Hooper Visual Organization Test, *GDS-15* Geriatric Depression Scale (15-item), *LBD* Lewy Body Dementia, *MMSE* Mini Mental State Examination, *MoCA* Montreal Cognitive Assessment, *M1* primary motor cortex, *N* number, *NMS* non-motor symptoms, *NMSS* Non-Motor Symptoms Scale, *NPI* Neuropsychiatric Inventory, *NR* not reported, *Oz* occipital cortex, *PCL* Probabilistic Classification Learning, *PD* Parkinson’s disease, *PSP* Progressive Supranuclear Palsy, *RCT* randomized controlled trial, *TMT* Trail Making Test, *trt* treatment, *WCST* Wisconsin Card Sorting Test, *WM* working memory test, *WMS* R Wechsler Memory Scale Revised

^a^Number of patients who completed the study and were analysed/number of included patients

^b^Type of outcome (scale), primary/secondary outcome of the study

^c^Significance is reported for between group (real vs. sham or different tDCS groups) analyses

^d^Study including patients with LBD + PD dementia

^e^Study including patients with PSP

#### Cognitive NMS

Cognitive outcomes were explored in 6 studies (primary, N = 1; secondary, N = 3; not specified, N = 2). Measures of global cognition did not change after real vs sham tDCS of the left DLPFC (N = 2) (Doruk et al. 2014; Cappiello et al. 2024), the left M1 (N = 1) (Simonetta et al. 2024) or the right posterior parietal cortex (N = 1) (Elder et al. 2019). One out of 3 studies, which explored single cognitive domains, found a significant effect of real vs sham left and right DLPFC on attention with delayed response at 1 month but no effect at the end of treatment (Doruk et al. 2014), and no effect on other domains to left DLPFC or left M1 and DLPFC anodal tDCS (Manor et al. 2021; Aksu et al. 2022).

#### Neuropsychiatric NMS

Depression was a secondary outcome in four studies and did not improve after left DLPFC or left M1, PFC and right posterior parietal cortex anodal tDCS (Benninger et al. 2010; Doruk et al. 2014; Elder et al. 2019; Cappiello et al. 2024). Anxiety was the secondary outcome of a single study that yielded negative findings for the left DPLFC anodal tDCS (Doruk et al. 2014). Psychosis (i.e., visual hallucinations) and apathy were the primary and secondary outcomes, respectively, of two studies, which yielded negative findings for the right posterior parietal cortex and left DLPFC anodal tDCS (Elder et al. 2019; Cappiello et al. 2024).

### Risk of bias

Overall, all the included studies were rated as raising some concerns (N = 26) or at high risk of bias (N = 8) due to several methodological issues, most commonly affecting the randomization process, deviation from intended interventions, and the selection of the reported results. Intention-to-treat (ITT)/modified-ITT analyses were missing in 26/34 reports (Fig. S1).

### Meta-analyses

Twenty-nine meta-analyses were carried out on the effect of NIBS on cognitive and neuropsychiatric NMS in patients with PD. We were unable to meta-analyse studies on tDCS and those on AP patients because of differences in brain targets and patient populations. For trials using rTMS, separate meta-analyses were performed for cognitive and neuropsychiatric NMS, different brain targets and different assessment timepoints (i.e., post-treatment, 1- or 3-month follow-up). As the number of studies included in the meta-analysis per outcome was low, moderator analyses and visual inspection of funnel plots for publication bias could not be performed (Borenstein et al. 2009). Detailed information on the specific effect sizes for each of the included studies and results of the meta-analyses are displayed graphically in the forest plots (Figs. S2–S15) and in Tables 3 and 4, respectively.

**Table 3:** Results of the meta-analyses on cognitive non-motor symptoms in Parkinson’s disease

	Outcome	K	N	Random-effect model results				Heterogeneity		
				MD/SMD	[95% CI]	Z	*p*	χ^2^	*p*	I^2^ (%)
	*Left DLPFC—excitatory TMS protocols (HF rTMS, iTBS)*									
*End of trt*	Global cognition	5	191	0.00	[− 0.49, 0.49]	0.02	0.99	11.03	0.03	64
	Attention	4	164	0.12	[− 0.19, 0.43]	0.75	0.46	2.10	0.55	0
	Memory	3	104	0.10	[− 0.37, 0.57]	0.42	0.68	2.89	0.24	31
	Language	3	104	0.04	[− 0.58, 0.66]	0.12	0.91	4.95	0.08	60
	Visuospatial	3	104	0.34	[− 0.05, 0.73]	1.69	0.09	0.11	0.95	0
	Executive function	3	129	− 0.00	[− 0.35, 0.34]	0.02	0.99	0.69	0.71	0
*1 mo FU*	Global cognition	4	153	− 0.09	[− 0.52, 0.34]	0.42	0.68	5.13	0.16	42
	Attention	2	69	− 0.09	[− 0.41, 0.23]	0.54	0.59	0.18	0.67	0
	Memory	2	69	0.11	[− 0.32, 0.53]	0.49	0.63	0.02	0.88	0
	Language	2	69	− 0.01	[− 0.36, 0.34]	0.07	0.95	0.15	0.69	0
	Visuospatial	2	69	0.16	[− 0.17, 0.48]	0.94	0.35	0.14	0.71	0
	Executive function	2	69	0.09	[− 0.31, 0.50]	0.45	0.66	0.01	0.92	0
*3 mos FU*	Global cognition	2	62	0.48	[− 0.03, 0.99]	1.84	0.07	0.95	0.33	0
	*Right DLPFC—inhibitory TMS protocols (LF rTMS)*									
*End of trt*	Global cognition	2	96	2.94	[2.31, 3.56]	9.22	** < 0.00001**	0.29	0.59	0
*3 mos FU*	Global cognition	2	96	3.47	[2.82, 4.13]	10.38	** < 0.00001**	0.69	0.41	0
	*Bilateral M1—excitatory TMS protocols (HF rTMS, iTBS)*									
*End of trt*	Global cognition	3	106	0.36	[− 1.87, 2.59]	0.32	0.75	5.06	0.08	60
*1 mo FU*	Global cognition	3	108	− 0.18	[− 1.92, 1.57]	0.20	0.84	3.12	0.21	36
*3 mos FU*	Global cognition	2	62	1.48	[− 1.39, 4.35]	1.01	0.31	0.15	0.70	0

*CI* confidence interval, *DLPFC* dorsolateral prefrontal cortex, *FU* follow-up, *HF* high frequency, *iTBS* intermittent theta burst stimulation, *K* number of studies, *MD* mean difference, *mo(s)* month(s), *M1* primary motor cortex, *N* number of participants, *rTMS* repetitive transcranial magnetic stimulation, *SMD* standardized mean difference, *trt* treatment

*P* values ≤ 0.05 are reported in bold type

**Table 4:** Results of the meta-analyses on neuropsychiatric non-motor symptoms in Parkinson’s disease

	Outcome	K	N	Random-effect model results				Heterogeneity		
				MD/SMD	[95% CI]	Z	*p*	χ^2^	*p*	I^2^ (%)
	*Left DLPFC—excitatory TMS protocols (HF rTMS, iTBS)*									
*End of trt*	Depression	5	154	− 0.55	[− 1.06, − 0.04]	2.12	**0.03**	8.76	0.07	54
	Anxiety	3	112	− 0.36	[− 0.73, 0.02]	1.87	0.06	0.75	0.69	0
	Apathy	3	79	0.93	[− 1.53, 3.38]	0.74	0.46	0.47	0.79	0
*1 mo FU*	Depression	4	130	0.30	[− 0.48, 1.08]	0.76	0.45	12.99	0.005	77
	Anxiety	3	112	− 0.23	[− 0.77, 0.32]	0.81	0.42	3.97	0.14	50
	Apathy	2	55	0.95	[− 3.95, 5.85]	0.38	0.70	1.82	0.18	45
	*Right DLPFC—inhibitory TMS protocols (LF rTMS)*									
*1 mo FU*	Depression	2	113	− 5.01	[− 7.53, − 2.49]	3.90	** < 0.0001**	0.02	0.90	0
	*Bilateral M1—excitatory TMS protocols (HF rTMS, iTBS)*									
*End of trt*	Depression	4	156	− 0.55	[− 1.07, − 0.02]	2.03	**0.04**	7.77	0.05	61
	Anxiety	3	112	− 0.75	[− 1.35, − 0.14]	2.42	**0.02**	4.78	0.09	58
*1 mo FU*	Depression	4	156	− 0.82	[− 1.69, 0.04]	1.87	0.06	19.07	0.0003	84
	Anxiety	3	112	− 1.02	[− 2.22, 0.19]	1.65	0.10	16.91	0.0002	88

*P* values ≤ 0.05 are reported in bold type

*CI* confidence interval, *DLPFC* dorsolateral prefrontal cortex, *FU* follow-up, *HF* high frequency, *iTBS* intermittent theta burst stimulation, *K* number of studies, *MD* mean difference, *mo(s)* month(s), *M1* primary motor cortex, *N* number of participants, *rTMS* repetitive transcranial magnetic stimulation, *SMD* standardized mean difference, *trt* treatment

### Effect of rTMS on cognitive NMS

#### Left DLPFC

Measures of global cognition, attention, memory, language, visuospatial, and executive functions did not differ for real vs sham excitatory HF rTMS or iTBS at the end of treatment, 1-month or 3-month follow-up (all *p* > 0.05; see Table 3). Heterogeneity was substantial for global cognition (I^2^ = 64%) and language (I^2^ = 60%) at the end of treatment, and moderate for memory (I^2^ = 30%) and global cognition (I^2^ = 42%) at the end of treatment and at 1-month follow-up, respectively.

#### Right DLPFC

Real inhibitory LF rTMS was superior to sham in ameliorating measures of global cognition at the end of treatment (MD = 2.94; 95% CI 2.31, 3.56; *p* < 0.00001); these effects were maintained at 3-month follow-up (MD = 3.47; 95% CI 2.82, 4.13; *p* < 0.00001). Heterogeneity was absent (I^2^ = 0%) for both timepoints.

#### Bilateral M1

Measures of global cognition, attention, memory, language, visuospatial, and executive functions did not differ for real vs sham excitatory HF rTMS or iTBS at the end of treatment, 1-month or 3-month follow-up (all *p* > 0.05; see Table 3). Heterogeneity was substantial (I^2^ = 60%) and moderate (I^2^ = 36%) for global cognition at the end of treatment, and 1-month follow-up, respectively.

### Effects of rTMS on neuropsychiatric NMS

#### Left DLPFC

Real excitatory HF rTMS or iTBS was superior to sham in reducing depressive symptoms at the end of treatment (SMD = − 0.55; 95% CI − 1.06, − 0.04; *p* = 0.03), but the effect was not maintained at 1-month follow-up (*p* = 0.45). A trend towards significance was found for real excitatory HF rTMS or iTBS vs sham in reducing anxiety at the end of treatment (MD = − 0.36; 95% CI − 0.73, 0.02; *p* = 0.06), but not at 1-month follow-up. Measures of apathy did not differ between real and sham groups at the end of treatment or 1-month follow-up (all *p* > 0.05; see Table 4). Heterogeneity was substantial (I^2^ = 54%) and considerable (I^2^ = 77%) for depression at the end of treatment and at 1-month follow-up, respectively, and moderate for anxiety (I^2^ = 50%) and apathy (I^2^ = 45%) at 1-month follow-up.

#### Right DLPFC

Real inhibitory LF rTMS was superior to sham in reducing depressive symptoms at 1-month follow-up (MD = − 5.01; 95% CI − 7.53, − 2.49; p < 0.0001). Heterogeneity was absent (I^2^ = 0%).

#### Bilateral M1

Real excitatory HF rTMS was superior to sham in reducing measures of depression and anxiety at the end of treatment (depression SMD = -0.55; 95% CI − 1.07, − 0.02; *p* = 0.04; anxiety SMD = − 0.75; 95% CI − 1.35, − 0.14; *p* = 0.02), but the effects were not maintained at 1-month follow-up (all *p* > 0.05; see Table 4). Heterogeneity was substantial for depression (I^2^ = 61%) and anxiety (I^2^ = 58%) at the end of treatment and became considerable (depression: I^2^ = 84%, anxiety: I^2^ = 88%) at 1-month follow-up.

### Sensitivity analyses

Sensitivity analyses were performed for meta-analyses where at least three studies were included, and either heterogeneity was statistically significant, or the effect size was not significant. Detailed results are displayed in Tables S3 and S4.

### Sensitivity analyses of the effects of rTMS on cognitive NMS

#### Left DLPFC

Heterogeneity was no longer significant at the end of treatment for global cognition, memory, and language after removing (He et al. 2021). However, the associated effect sizes remained not significant. Similarly, heterogeneity for global cognition at 1-month follow-up decreased to zero after removing (Brys et al. 2016), but the overall effect size remained not significant (Table S2).

#### Bilateral M1

Heterogeneity was no longer significant for global cognition after removing (Khedr et al. 2020) and (Brys et al. 2016) at the end of treatment and 1-month follow-up, respectively. However, the associated effect sizes remained not significant (Table S2).

### Sensitivity analyses of the effects of rTMS on neuropsychiatric NMS

#### Left DLPFC

After excluding (Brys et al. 2016), the overall effect size for depression at the end of treatment became significant, indicating that excitatory HF rTMS/iTBS of the left DLPFC was superior to sham in reducing depression, and heterogeneity decreased to moderate. Excluding (Jiang et al. 2023) only resulted in a reduction in heterogeneity, which became low. The effect size for anxiety at the end of treatment became significant after removing (Trung et al. 2019), indicating that excitatory HF rTMS or iTBS targeting the left DLPFC was superior to sham in reducing anxiety, and heterogeneity remained not significant. In contrast, removal of (Jiang et al. 2023) caused no changes of heterogeneity, which remained not significant. For apathy at the end of treatment, no changes in the significance of the effect size or in the magnitude of heterogeneity were observed after excluding (Trung et al. 2019) (Table S3).

For depression at 1-month follow-up, the effect size became significant after excluding (Brys et al. 2016), indicating that the sham condition was superior to real excitatory HF rTMS/iTBS in reducing depressive symptoms, and the heterogeneity became moderate. For anxiety at 1-month follow-up, no changes were observed in the significance of the effect size or the magnitude of heterogeneity after excluding (Jiang et al. 2023) (Table S3).

#### Bilateral M1

The overall effect sizes for depression at the end of treatment and 1-month follow-up became significant and heterogeneity was not significant and moderate after removal of (Brys et al. 2016), indicating that excitatory HF rTMS/iTBS targeting bilateral M1 was superior to sham in reducing depressive symptoms. After removing the same study (Brys et al. 2016), the overall effect size for anxiety at the end of treatment and 1-month follow-up became significant and heterogeneity was not significant and moderate after removing the same study (Table S3).

## Discussion

The present study collected evidence on NIBS for the treatment of cognitive and neuropsychiatric NMS in PD and AP through a systematic search and meta-analytic approach. Thirty-four RCTs investigating the efficacy of rTMS and tDCS for cognitive and some neuropsychiatric NMS (i.e., depression, anxiety, apathy, psychosis) were identified, while data on other NIBS approaches (e.g., LIFU, TPS) were lacking. Moreover, we found no studies on NIBS for the treatment of other common and clinically relevant neuropsychiatric NMS (i.e., ICDs, impulsivity, anhedonia, akathisia).

The meta-analytic review found that PD patients who received real rTMS/iTBS did not differ significantly from those undergoing sham treatment in cognitive or neuropsychiatric outcomes at the end of treatment or 1-to-3-month follow-up, except for depression. Sensitivity analyses showed medium-to-large effect size of real HF rTMS/iTBS targeting the left DLPFC on depression at the end of treatment that was not maintained at 1-month follow-up, and small-to-medium effect of real HF rTMS/iTBS on the left DLPFC on anxiety at the end of treatment. The meta-analysis of two studies with no heterogeneity found real inhibitory LF rTMS on the right DLPFC to be superior to sham for depression with large effect size up to 3-month follow-up. Medium-to-large and large effect sizes were documented by the sensitivity analyses for HF rTMS targeting bilateral M1 on depression at the end of treatment and 1-month follow-up, respectively.

Additionally, results from qualitative synthesis highlighted that targeting other brain areas (e.g., parietal cortices, insula, SMA), using different rTMS protocols that combined stimulation of multiple brain areas, or applying tDCS did not improve cognitive and neuropsychiatric NMS to NIBS. Data on the use of NIBS for treating cognitive and neuropsychiatric NMS associated with AP were less consistent, being limited to significant effects for HF rTMS targeting the left DLPFC on depression and anxiety in patients with MSA (Pan et al. 2022).

Our results are consistent with those of a recently published meta-analysis (Giustiniani et al. 2025), which found no evidence of efficacy of real rTMS/tDCS over sham on PD-related cognitive NMS, regardless of targeted brain area, stimulation type, protocol, and treatment duration (i.e., single vs. multiple sessions). Cognitive NMS in PD and AP are a major therapeutic challenge, in that they are supposed to reflect multiple underlying pathologies (i.e., amyloid-β deposition, tau neurofibrillary tangles, α-synuclein accumulation) in several brain areas (e.g., frontal, parietal, and temporal cortices), which lead to dysfunction of multiple neurotransmitter (i.e., dopaminergic, serotonergic, noradrenergic, cholinergic, glutamatergic) pathways (Mantovani et al. 2024b). The complex interplay of different neuropathologies and neurotransmitter system dysfunction may lead to network failure, requiring more precise NIBS targeting based on functional connectivity analysis tailored to specific neuropsychiatric symptoms (Cash and Zalesky 2024). Encouraging data are emerging from NIBS to improve cognitive function or slow cognitive decline in patients with Alzheimer’s disease (AD) that share amyloid-β deposition and tau neurofibrillary tangles with PD-related cognitive decline. Following a precision medicine approach that combines neurophysiological and behavioral data, very promising results have been obtained by targeting key hubs of higher cognitive networks, such as the frontal-parietal and the default mode networks (Koch et al. 2024). A similar approach warrants testing in PD to improve cognitive NMS.

Our data on neuropsychiatric NMS align with those of a previous meta-analysis (Zheng et al. 2022), which found evidence of efficacy for real M1/DLPFC rTMS on PD-related depression and anxiety, but not on apathy, regardless of stimulation parameters. This is not surprising as the current guidelines report left DLPFC HF rTMS as definitely (level A) and probably (level B) effective non-pharmacological treatment for depression in the general population and in PD, respectively (Lefaucheur et al. 2020). The finding that inhibitory LF rTMS on the right DLPFC improved depression in PD is in keeping with data on drug-resistant depression in the general population that yielded probably (level B) effectiveness for this TMS approach/target (Lefaucheur et al. 2020). The improvement of depression following HF rTMS of M1 in PD is a more counterintuitive finding, which has been ascribed to the frontal longitudinal system, a set of fibers mediating the axonal connectivity of the prefrontal-premotor cortices and serving cognitive-motor functions (Komaitis et al. 2019). In addition, a previous work combining results from a meta-analysis with resting-state fMRI data reported a positive correlation between changes in M1 and regions-of-interest in depressive disorders, supporting the view that targeting M1 may have antidepressant effects (Zhang et al. 2020).

Not surprisingly, our findings suggest that anxiety may also benefit from M1/DLPFC rTMS in PD, although to a minor extent than depression. Anxiety and depression often co-occur in PD, and they partially share a pathophysiological network involving the prefrontal and orbitofrontal cortices, anterior cingulate cortex, insula, amygdala, and ventral tegmental area (Zhao et al. 2025).

The isolated finding of a significant effect for left DLPFC HF rTMS on depression and anxiety in MSA (Pan et al. 2022) is promising but requires further confirmation by other studies.

Negative findings have been reported for apathy treated with HF rTMS over the left DLPFC. Pathophysiological data suggest that apathy in PD may be related to functional and structural changes in subcortical or deep cortical regions (e.g., nucleus accumbens) within the reward system (Morris et al. 2023). Therefore, classical NIBS techniques (i.e., rTMS, tDCS) may not be the best therapeutic approaches to apathy as they target superficial brain structures. LIFU may be a viable alternative to be tested in future studies, given its ability to reach deeper brain areas up to the basal ganglia and thalamus.

A single study yielded negative findings on psychosis (visual hallucinations) in PD-dementia and DLB to right parietal tDCS (Elder et al. 2019), suggesting that other targets should be tested to address this common NMS.

Notwithstanding their high prevalence and impact on the quality of life of PD patients and caregivers, we found no data on NIBS to treat other neuropsychiatric NMS, e.g., ICDs. Despite the lack of therapeutical choices for ICDs in PD (Mantovani et al. 2023), promising despite preliminary results have been reported for HF rTMS targeting the left DLPFC in behavioral addictions in the general population, a condition that is supposed to share pathophysiology with PD-related ICDs (Ekhtiari et al. 2019; Zucchella et al. 2020). The alteration of top-down control processing, monitored by a complex mesolimbic (e.g., ventrolateral prefrontal cortex, anterior cingulate cortex), prefrontal and parietal network is considered a common hallmark of ICDs and behavioral addictions (Niendam et al. 2012; Martini et al. 2020). Future studies should test the feasibility of using either rTMS or TDCS to modulate cortical areas or LIFU to target deep mesolimbic structures for ICDs in PD.

### Strength and limitations

The strengths of this report are the thorough search criteria that included all cognitive and neuropsychiatric NMS associated with both PD and AP, and less common NIBS techniques (e.g., tACS, tRNS, LIFU, TPS), the strict criteria for inclusion of studies (i.e., only studies with a control group) and for defining efficacy of NIBS (i.e., only if confirmed by between-groups comparison). Other strengths include different quantitative analyses according to stimulation targets and effect on brain excitability (i.e., excitatory left DLPFC, inhibitory right DLPFC, and bilateral M1 rTMS), the qualitative analysis of studies on AP and tDCS, and a roadmap for future trials.

The main limitation results from the high variability of targets, NIBS protocols and outcomes and the small sample sizes of included studies that resulted in partially underpowered meta-analyses and the lack of clear effects of NIBS on cognitive/neuropsychiatric NMS in PD. We also acknowledge the limited number of studies on AP that impeded a quantitative synthesis of the results. Even if a formal GRADE assessment was not performed, the low-to-moderate overall certainty of the evidence, and the potential influence of outliers as indicated by the results of sensitivity analyses, suggest that the positive effects of rTMS on depression and anxiety should be interpreted with caution and as still largely exploratory. Furthermore, the small-study effect could not be excluded because publication bias could not be assessed. Several methodological factors may have contributed to this not conclusive finding.

### A roadmap for improving the quality of RCTs on NIBS for cognitive and neuropsychiatric NMS in PD

We propose a roadmap to address the issues and limitations that affect current literature and may have contributed to the overall not conclusive findings on the role of NIBS for cognitive/neuropsychiatric NMS in PD and AP.

First, some studies targeted brain areas lacking neurobiological evidence. M1 was one of the most targeted brain areas, despite this region is not reported to contribute to the pathophysiology of cognitive and neuropsychiatric NMS in PD and AP. Several studies targeted the DLPFC with a one-size-fits-all approach to NMS, which are varied and related to different brain changes and pathophysiological abnormalities. Animal models of cognitive/neuropsychiatric NMS in PD and AP may better elucidate the underlying pathophysiology and provide more robust brain targets for testing in RCTs in PD/AP patients. Functional connectivity analyses have been proposed to tailor NIBS treatment (Cash and Zalesky 2024), and they may offer important information in PD/AP patients. Combining functional neuroimaging and neurophysiological data with those from systematic reviews and meta-analyses can better define brain functional “fingerprints” of a given NMS that is both neurobiologically plausible and personalized for a given patient. Neuroimaging, neurophysiological, and biofluid biomarkers of NMS that are becoming more available may offer neuropathological and neuropharmacological information to stratify patients and predict response to NIBS (Mantovani et al. 2024b).

Second, we found several underpowered studies with high heterogeneity in NIBS parameters, total number of stimulation sessions, and timing of follow-up. Cognitive/neuropsychiatric NMS were often secondary outcome measures, or whether they were primary or secondary outcomes was not defined, with no a priori sample size assessment. The severity of cognitive/neuropsychiatric NMS was not detailed in most studies, leading to the potential inclusion of PD/AP patients without clinically significant NMS and to a ceiling effect that may have biased the results (Giustiniani et al. 2025). Future RCTs with multicenter design to recruit larger samples of PD/AP patients, based on power analyses focused on cognitive and neuropsychiatric NMS-related outcomes, will overcome some of these methodological limitations. The treatment duration should be long enough to result in robust neuromodulation, with accelerated NIBS protocols and/or booster sessions to maximize long-term effects that should be assessed with adequate follow-ups.

Third, there are other methodological considerations to improve the design of future studies. TBS has been proposed as an accelerated NIBS technique, but it appears to have limited efficacy on PD/AP-related NMS. The modulatory effects of TBS have been demonstrated to vary substantially between and within individuals, with most not being significantly different from sham-TBS (Ozdemir et al. 2021). Measures of cortical excitation and inhibition may predict response to TBS in depression (Dhami et al. 2023). Assessment of responders/non-responders to specific NIBS protocols may help select patients with a higher chance of response.

Fourth, LIFU or deep TMS may target subcortical structures in the mesencephalon and the basal ganglia, which are affected from the first stages of PD and AP and underlie some cognitive/neuropsychiatric NMS, e.g., apathy, increase the efficacy of NIBS compared to rTMS and tDCS.

Fifth, in clinical settings, most patients are treated with a multimodal approach including drugs, cognitive rehabilitation, and psychotherapy according to the target NMS. Future NIBS studies should combine these pharmacological and non-pharmacological approaches to explore if they have an additive effect and may prolong the effects of NIBS (Mantovani et al. 2024a).

**Fig. 2:**
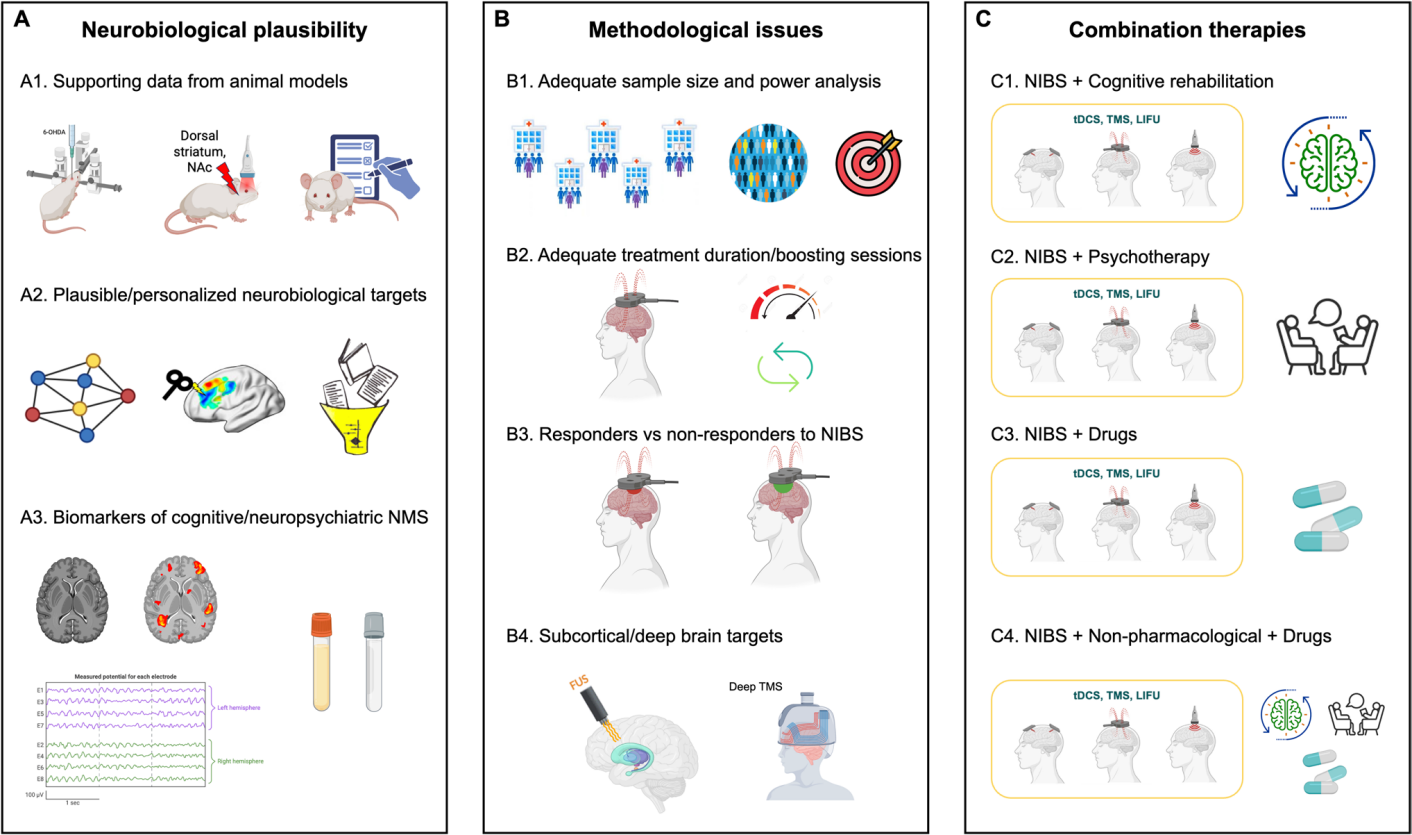
A roadmap for improving NIBS studies for the treatment of cognitive and neuropsychiatric NMS in PD. Panel **A**. Future RCTs should target brain areas with a neurobiological plausibility. Animal models of cognitive and neuropsychiatric NMS in PD and AP may better elucidate underlying pathophysiology and offer more robust brain targets to be tested in RCTs in PD/AP patients (A1). Combining results of functional connectivity analyses in patients according to their NMS, and their modulation by NIBS with data from systematic reviews and meta-analyses can offer plausible and personalized brain functional “fingerprints” of cognitive and neuropsychiatric NMS (A2). Neuroimaging, neurophysiological, and biofluid biomarkers of NMS may offer information on the underlying neuropathological and neuropharmacological changes to better stratify patients and inform response to NIBS treatment (A3). Panel **B**. Multicenter NIBS RCTs can recruit larger samples of PD/AP patients according to a power analyses focused on cognitive and neuropsychiatric NMS-related outcomes (B1). Adequate treatment duration (i.e., long enough to result in a robust neuromodulation) and the use of accelerated NIBS protocols and/or booster sessions may maximize long-term effects, and help detecting clinically significant differences in cognitive and neuropsychiatric NMS in PD/AP after NIBS (B2). Preliminary assessment of responders/non-responders to specific NIBS protocols may result in the selection of patients with a higher chance of improving NMS outcomes (B3). Targeting subcortical or deep brain areas using LIFU or deep TMS may increase the efficacy of NIBS for those cognitive or neuropsychiatric NMS (e.g., apathy) that are associated to damage or dysfunction of subcortical structures (B4). Panel C. Future RCTs should test personalized multimodal treatment approaches that combine NIBS with other non-pharmacological interventions, e.g., cognitive rehabilitation for cognitive NMS (C1), cognitive-behavioral psychotherapy for neuropsychiatric NMS (C2), drugs that can boost the effect of NIBS on NMS (C3) or the combination of NIBS with pharmacological and non-pharmacological treatments (C4). *AP* atypical parkinsonism, *LIFU* low-intensity focused ultrasound, *NIBS* non-invasive brain stimulation, *NMS* non-motor symptoms, *PD* Parkinson’s disease, *RCTs* randomized controlled trials, *TMS* transcranial magnetic stimulation, *tDCS* transcranial direct current stimulation. This figure was partially created with Biorender.com (; BioRender 2025)

To sum up, future RCTs could be improved by (a) pathophysiology-guided target selection, (c) well-powered multicentre trials with appropriate primary outcomes and follow-up, (c) inclusion of biomarkers of treatment response, (d) exploration of deep-target NIBS, and (e) multimodal approaches (Fig. 2).

## Conclusions

Current data are insufficient to support routine NIBS for cognitive NMS in PD or AP, while excitatory TMS protocols (i.e., HF rTMS, iTBS) targeting the left DLPFC showed promising but still preliminary effects for depression and anxiety in PD. Studies involving longer-term follow-ups are required to confirm long-lasting benefits. No studies were found on NIBS for impulsivity, ICDs, anhedonia, or akathisia in PD/AP. The proposed roadmap might help overcome the limitations of current literature evidence on this topic.

## Supplementary Information

Below is the link to the electronic supplementary material.

Supplementary file1 (PDF 5987 KB)

## Data Availability

The datasets generated during and/or analyzed during the current study are available from the corresponding author on reasonable request.
