# Supplementary material for: Non-invasive brain stimulation for treating cognitive and neuropsychiatric non-motor symptoms in Parkinson’s disease and atypical parkinsonism: a systematic review and meta-analysis of randomized controlled trials
Source: J Neural Transm (Vienna). 2026 Mar 5;133(4):705–29. doi: 10.1007/s00702-026-03127-x (PMC13149570; doi:10.1007/s00702-026-03127-x)
Supplement: Supplementary file 1 — Supplementary file1 (PDF 5987 KB) [file 702_2026_3127_MOESM1_ESM.pdf]

SUPPLEMENTARY MATERIAL

Non-invasive brain stimulation for treating cognitive and neuropsychiatric non-motor symptoms in Parkinson's disease and atypical parkinsonism: a systematic review and meta-analysis of randomized controlled trials

Elisa Mantovani[1,^], Eleonora Bertoncello[1], Mirko Filippetti[1], Alessandro Picelli[1], Michele Tinazzi[1], Stefano Tamburin[1,^]

[1]Department of Neurosciences, Biomedicine and Movement Sciences, University of Verona, Piazzale L.A. Scuro 10, I-37134, Verona, Italy

[^]**Corresponding authors.** Elisa Mantovani, MPsych, PhD, Department of Neurosciences, Biomedicine and Movement Sciences, Neurology Section, University of Verona, Piazzale Scuro 10, I-37134 Verona, Italy; Tel.: +39-347-630-5905; Fax: +39-045-802-7276; Email address: elisa.mantovani@univr.it; ORCID: 0000-0003-3717-7697.

Stefano Tamburin, MD, PhD, Section of Neurology, Department of Neurosciences, Biomedicine and Movement Sciences, University of Verona, Piazzale Scuro 10, I-37134 Verona, Italy. Tel.: +39-347-523-5580; fax: +39-045-802-7276; email: stefano.tamburin@univr.it; ORCID: 0000-0002-1561-2187.

Summary:

Search strategies

Supplementary table 1

Supplementary table 2

Supplementary table 3

Supplementary table 4

Supplementary figure S1

Supplementary figures S2-S15

Supplementary figure legends

Search strategies

Search strategy for PubMed

("non invasive brain stimulation" OR "non-invasive brain stimulation" OR NIBS OR transcranial magnetic stimulation OR TMS OR "repetitive transcranial magnetic stimulation" OR rTMS OR "theta burst stimulation" OR TBS OR transcranial direct current stimulation OR tDCS OR transcranial alternating current stimulation OR tACS OR transcranial random noise stimulation OR tRNS OR "deep transcranial magnetic stimulation" OR "deep TMS" OR neuromodulation OR "transcranial focused ultrasound" OR tFUS OR "low intensity focused ultrasound" OR "low-intensity focused ultrasound" OR LIFU OR "transcranial pulse stimulation" OR "TPS") NOT (neurotransmitter agents OR deep brain stimulation OR DBS OR "magnetic resonance imaging guided focused ultrasound" OR "magnetic resonance guided focused ultrasound" MRgFUS OR "high intensity focused ultrasound" OR HIFU) AND (Parkinson disease OR "atypical parkinsonism*" OR lewy body disease OR "dementia with lewy bodies" OR " lewy body dementia" OR supranuclear palsy, progressive OR corticobasal degeneration OR "corticobasal syndrome" OR multiple system atrophy) AND ("non motor symptoms" OR "neuropsychiatric non motor symptoms" OR cognit* OR cognitive dysfunction OR "mild cognitive impairment" OR dementia OR "cognitive impairment" OR anxiety OR depression OR apathy OR akathisia OR anhedonia OR "impulse control disorders" OR "impulsive compulsive behaviors" OR ICD OR ICB OR psychosis)

Search strategy for the Cochrane CENTRAL Register of Controlled Trials

("non invasive brain stimulation" OR "non-invasive brain stimulation" OR NIBS OR transcranial magnetic stimulation OR TMS OR "repetitive transcranial magnetic stimulation" OR rTMS OR "theta burst stimulation" OR TBS OR transcranial direct current stimulation OR tDCS OR transcranial alternating current stimulation OR tACS OR transcranial random noise stimulation OR tRNS OR "deep transcranial magnetic stimulation" OR "deep TMS" OR neuromodulation OR "transcranial focused ultrasound" OR tFUS OR "low intensity focused ultrasound" OR "low-intensity focused ultrasound" OR LIFU OR "transcranial pulse stimulation" OR "TPS") NOT (neurotransmitter agents OR deep brain stimulation OR DBS OR "magnetic resonance imaging guided focused ultrasound" OR "magnetic resonance guided focused ultrasound" MRgFUS OR "high intensity focused ultrasound" OR HIFU) AND (Parkinson disease OR atypical NEXT parkinsonism* OR lewy body disease OR "dementia with lewy bodies" OR " lewy body dementia" OR supranuclear palsy, progressive OR corticobasal degeneration OR "corticobasal syndrome" OR multiple system atrophy) AND ("non motor symptoms" OR "neuropsychiatric non motor symptoms" OR cognit* OR cognitive dysfunction OR "mild cognitive impairment" OR dementia OR "cognitive impairment" OR anxiety OR depression OR apathy OR akathisia OR anhedonia OR "impulse control disorders" OR "impulsive compulsive behaviors" OR ICD OR ICB OR psychosis): ti,ab,kw" (Word variations have been searched)

EMBASE (via Ovid) search strategy

	Search terms
1	'non invasive brain stimulation'.tw.
2	NIBS.tw.
3	(magnetic stimulation, transcranial or magnetic stimulations, transcranial or stimulation, transcranial magnetic or stimulations, transcranial magnetic or transcranial magnetic stimulation or transcranial magnetic stimulation, repetitive or transcranial magnetic stimulations).mp.
4	TMS.tw.
5	rTMS.tw.
6	'theta burst stimulation'.tw.
7	TBS.tw.
8	(anodal stimulation transcranial direct current stimulation or anodal stimulation tdcs or anodal stimulation tdcss or cathodal stimulation transcranial direct current stimulation or cathodal stimulation tdcs or cathodal stimulation tdcss or electrical stimulation, transcranial or electrical stimulations, transcranial or repetitive transcranial electrical stimulation or stimulation, transcranial electrical or stimulation tdcs, anodal or stimulation tdcs, cathodal or stimulation tdcss, anodal or stimulation tdcss, cathodal or stimulations, transcranial electrical or transcranial alternating current stimulation or transcranial direct current stimulation or transcranial electrical stimulation or transcranial electrical stimulations or transcranial random noise stimulation or tdcs or tdcs, anodal stimulation or tdcs, cathodal stimulation or tdcss, anodal stimulation or tdcss, cathodal stimulation).mp.
9	tACS.tw.
10	tRNS.tw.
11	'deep transcranial magnetic stimulation'.tw.
12	'deep TMS'.tw.
13	'transcranial focused ultrasound'.tw.
14	tFUS.tw.
15	'low intensity focused ultrasound'.tw.
16	LIFU.tw.
17	'transcranial pulse stimulation'.tw.
18	TPS.tw.
19	neuromodulation.tw.
20	#1 OR #2 OR #3 OR #4 OR #5 OR #6 OR #7 OR #8 OR #9 OR #10 OR #11 OR #12 OR #13 OR #14 OR #15 OR #16 OR #17 OR #18 OR #19
21	(brain stimulation, deep or brain stimulations, deep or deep brain stimulation or deep brain stimulations or stimulation, deep brain or stimulations, deep brain).mp.
22	DBS.tw.
23	#21 OR #22
24	#20 NOT #23
25	Parkinson$.tw. or Parkinson Disease/
26	paralysis agitans.tw.

**27**	Primary Parkinsonism.tw.
**28**	Lewy Body Disease/ or (Supranuclear Palsy, Progressive/ or Multiple System Atrophy/) or Corticobasal Degeneration/
**29**	‘Atypical Parkinsonism’.mp
**30**	#25 OR #26 OR #27 OR #28 OR #29
**31**	'non motor symptom$'.tw.
**32**	'neuropsychiatric non motor symptom$'.tw.
**33**	cognit$.tw.
**34**	(cognitive decline or cognitive declines or cognitive disorder or cognitive disorders or cognitive dysfunction or cognitive dysfunctions or cognitive impairment or cognitive impairment, mild or cognitive impairments or cognitive impairments, mild or decline, cognitive or declines, cognitive or deterioration, mental or deteriorations, mental or disorder, cognitive or disorders, cognitive or dysfunction, cognitive or dysfunctions, cognitive or impairment, cognitive or impairment, mild cognitive or impairments, cognitive or impairments, mild cognitive or mental deterioration or mental deteriorations or mild cognitive impairment or mild cognitive impairments).mp.
**35**	MCI.tw.
**36**	'PD-MCI'.tw.
**37**	dementia.tw.
**38**	Anxiety/
**38**	Depression/
**39**	Apathy/
**40**	Anhedonia/
**41**	akathisia.tw.
**42**	exp "Disruptive, Impulse Control, and Conduct Disorders"/
**43**	'Impulse control disorder*'.tw.
**44**	'Impulsive compulsive behavio?r*'.tw.
**45**	ICD.tw.
**46**	ICB.tw.
**47**	Gambling.tw. or Gambling/
**48**	Walkabout.tw.
**49**	Hypersexuality.tw.
**50**	Sexual behavio?r.tw. or Sexual Behavior/
**51**	Punding.tw.
**52**	Hobbyism.tw.
**53**	'compulsive shopping'.tw.
**54**	'Binge eating'.tw.
**55**	'Compulsive eating'.tw. or Food Addiction/
**56**	'dopamine dysregulation syndrome'.tw.

**57**	#31 OR #32 OR #33 OR #34 OR #35 OR #36 OR #37 OR #38 OR #39 OR #40 OR #41 OR #42 OR #43 OR #44 OR #45 OR #46 OR #47 OR #48 OR #49 OR #50 OR #51 OR #52 OR #53 OR #54 OR #55 OR #56
**58**	#24 AND #30 AND #57

Additional searches: clinical trial registries

Source	Terms and limits
ClinicalTrials.gov	**Condition:** Parkinson Disease, Dementia with Lewy Bodies, Multiple System Atrophy, Progressive Supranuclear Palsy, Corticobasal Degeneration<br>**Other terms:** Mild cognitive impairment, Dementia, Cognitive Impairment, Impulse Control Disorders, Impulsive Compulsive Behaviors, Anxiety, Depression, Apathy, Akathisia, Anhedonia, Psychosis<br>**Intervention/treatment:** Non-Invasive Brain Stimulation, Transcranial Magnetic Stimulation, Transcranial Direct Current Stimulation, Transcranial Alternating Current Stimulation, Transcranial Random Noise Stimulation, Low Intensity Focused Ultrasound, Transcranial Pulse Stimulation<br>**Study status:** All status<br>**Phases:** 2,3,4<br>**Study type:** Interventional<br>**Study results:** With results<br>**Funder type:** Any

Table S1. Clinical data of populations with Parkinson’s disease in the included studies

Ref.	Sample (N, sex, age)		PD duration		H-Y		UPDRS III		LEDD (total)		Frequency/severity of cognitive NMS		Frequency/severity of neuropsychiatric NMS	
	Real	Sham	Real	Sham	Real	Sham	Real	Sham	Real	Sham	Active	Sham	Active	Sham
Okabe et al., 2003	N = 85 (M: 46, F: 37; age: 67.2 ± 8.2)		O1 = 8.8 ± 6.4<br>M1 = 8.8. ± 5.1	N = 8.0 ± 5.4	O1 = 2.95 ± 0.83<br>M1 = 3.11 ± 0.92	2.92 ± 0.83	O1 = 26.7 ± 11.8<br>M1 = 26.1 ± 16.3	22.3 ± 12.6	NR	NR	NA	NA	HAM-D (O1) = 7.4 ± 1.3<br>HAM-D (M1) = 10.6 ± 1.3	HAM-D = 7.4 ± 1.4
Hamada et al., 2008	N = 55 (M: 29, F: 26; age: 65.3 ± 8.9)	N = 43 (M: 25; F: 18; age: 67.4 ± 8.5)	8.1 ± 4.2	7.8 ± 6.7	2.8 ± 0.6	2.9 ± 0.7	23.0 ± 9.7	25.8 ± 13.5	NR	NR	NA	NA	HAM-D = 5.5 ± 4.8	HAM-D = 7.5 ± 5.6
Arias et al., 2010	N = 9	N = 9	NR	NR	NR	NR	NR	NR	NR	NR	NA	NA	HAM-D = 17.1 ± 4.6	HAM-D = 14.5 ± 5.4
Benninger et al., 2010	N = 13 (M: 9, F: 4; age: 63.6 ± 9.0)	N = 12 (M: 7, F: 5; age: 64.2 ± 8.8)	10.6 ± 7.1	9.1 ± 3.3	2.5 ± 0.1	2.4 ± 0.2	22.2 ± 8.7	17.5 ± 8	1024.3 ± 541.5	1287.7 ± 808.8	NA	NA	BDI = 10.4 ± 11.3	BDI = 7 ± 4.2
Pal et al., 2010	N = 12 (M: 6; F: 6; age: 66.6 ± 3.1)	N = 10 (M: 5, F: 5; age: 66.0 ± 4.3)	6.1 ± 1.9	6.8 ± 1.9	2.0 ± 0.5*	2.0 ± 0.5*	23.5 ± 12.0*	21.5 ± 15.0*	300 ± 144.3	325 ± 173.2	MMSE = 28.5 (1.5)*	MMSE = 29.0 (2.5)*	Mild dep = 7<br>Moderate dep = 5<br>BDI = 9.0 ± 4.5*<br>MADRS = 11.5 ± 13.5*	Mild dep = 6<br>Moderate dep = 4<br>BDI = 9.5 ± 6.0*<br>MADRS = 12.0 ± 14.0*
Benninger et al., 2012	N = 13 (M: 11, F: 2; age: 64.5 ± 9.1)	N = 13 (M: 9, F: 4; age: 63.7 ± 8.3)	8.6 ± 4.1	9.3 ± 6.8	2.4 ± 0.2	2.5 ± 0.3	32.08 ± 2.5	30.0 ± 2.6	861.0 ± 436.0	949.0 ± 677.0	FAB = 16.15 ± 0.5	FAB = 17.1 ± 0.6	BDI = 9.5 ± 1.8	BDI = 9.3 ± 1.7
ReStore, 2012 (NCT00955032)	N = 16 (M: 14, F: 2; age: 63.8 ± 7.2)	N = 8 (M: 5, F: 3; age: 72.8 ± 5.7)	NR	NR	NR	NR	NR	NR	NR	NR	NR	NR	NR	NR
Shirota et al., 2013	LF (N = 36; M: 14, F: 22; age: 68.8 ± 7.6)<br>HF (N = 44; M: 12, F: 22; age: 67.9 ± 8.4)	N = 36 (M: 19, F: 17; age: 65.7 ± 8.5)	LF = 8.5 ± 7.3<br>HF = 7.8 ± 6.6	7.6 ± 4.4	LF (2/3/4) = 10/21/5<br>HF (2/3/4) = 9/21/4	2 = 10<br>3 = 21<br>4 = 5	NR	NR	LF = 458.5 ± 256.0<br>HF = 454.6 ± 212.3	483.8 ± 244.5	NR	NR	NR	NR

Doruk et al., 2014	N = 18 (M: 12, F. 6; age: 61.0 ± 8.0)		NR	NR	NR	NR	NR	NR	NR	NR	NR	NR	NR	NR
Brys et al., 2016	M1 + DLPFC (N = 20; M: 11, F: 9; age: 64.9 ± 8.0)<br>M1 (N = 14; M: 9, F: 5; age: 59.6 ± 12.6)<br>DLPFC (N = 12; M: 6, F: 6; age: 64.6 ± 12.3)	N = 15 (M: 11, F: 4; age: 64.0 ± 7.4)	M1 + DLPFC = 7.3 ± 5.6<br>M1 = 8.4 ± 5.2<br>DLPFC = 7.7 ± 4.2	4.5 ± 2.2	M1 + DLPFC (2/2.5/3/4) = 8/7/4/1<br>M1 (2/2.5/3/4) = 7/6/1/0<br>DLPFC (2/2.5/3/4) = 4/2/3/3	2 = 7<br>2.5 = 6<br>3 = 2<br>4 = 0	M1 + DLPFC = 32.3 ± 8.9<br>M1 = 33.1 ± 7.8<br>DLPFC = 32.8 ± 10.7	28.9 ± 6.4	NR	NR	NR	NR	M1 + DLPFC = 60%[#]<br>M1 = 71.4%[#]<br>DLPFC = 66.7%[#]<br>HAM-D (M1+DLPFC) = 15.2 ± 6.0<br>BDI-II (M1+DLPFC) = 22.9 ± 12.1<br>HAM-D (M1) = 16.7 ± 3.9<br>BDI-II (M1) = 18.5 ± 8.3<br>HAM-D (DLPFC) = 13.8 ± 4.6<br>BDI-II (DLPFC) = 21.7 ± 11.9	60%[#]<br>HAM-D = 14.1 ± 3.7<br>BDI-II = 19.0 ± 8.0
Makkos et al., 2016	N = 23 (M: 13, F: 10; age: 66.7 ± 3.7)	N = 21 (M: 11, F: 10; age: 66 ± 2.3)	6.2 ± 2.0	5.5 ± 1.7	1 = 2<br>2 = 15<br>3 = 4<br>4 = 2	1 = 2<br>2 = 12<br>3 = 4<br>4 = 3	28.5 ± 8.7	28.5 ± 7.5	592.5 ± 164.5	535 ± 144.3	MMSE = 29.0 ± 1.6<br>MoCA = 25.0 ± 5.5	MMSE = 28.7 ± 2.4<br>MoCA = 21.7 ± 4.8	Mild dep = 13<br>Moderate dep = 10<br>MADRS = 16.3 ± 6.3<br>BDI = 11.7 ± 10.3	Mild dep = 12<br>Moderate dep = 9<br>MADRS = 14.7 ± 4.0<br>BDI = 12.0 ± 4.0
Shin et al., 2016	N = 10 (M: 6, F: 4; age: 68.7 ± 7.8)	N = 8 (M: 2, F: 6; age: 66.7 ± 7.2)	12.3 ± 9.5	6.3 ± 3.8	2 = 7<br>3 = 3	2 = 5<br>3 = 3	18.5 ± 9.5	17.5 ± 1.7	903.2 ± 372.3	747.6 ± 289.0	NA	NA	All patients had a diagnosis of MDD	All patients had a diagnosis of MDD
Buard et al., 2018	N = 22 (M: 16, F: 6; age: 67.4 ± 7.2)	N = 24 (M: 17, F: 7; age: 69.5 ± 8.0)	NR	NR	1 = 5<br>2 = 8<br>2.5 = 7<br>3 = 2	1 = 1<br>1.5 = 1<br>2 = 10<br>2.5 = 7<br>3 = 5	23.5 ± 8.8	25.7 ± 8.4	690.1 ± 481.7	514.3 ± 309.5	All patients were PD-MCI<br>CDR = 0.48 ± 0.19<br>MoCA = 24.6 ± 3.9	All patients were PD-MCI<br>CDR = 0.48 ± 0.19<br>MoCA = 25.3 ± 2.8	HADS-D = 5.6 ± 3.1<br>HADS-A = 5.7 ± 3.5	HADS-D = 4.6 ± 2.7<br>HADS-A = 6.0 ± 3.7
Cohen et al., 2018	N = 21 (M: 17, F: 4; age: 64.4 ± 6.8)	N = 21 (M: 15, F: 6; age: 66.8 ± 8.1)	4.7 ± 3.4	5.6 ± 3.7	2.1 ± 0.25	2.1 ± 0.25	NR	NR	255.6 ± 228.1	435.6 ± 321.0	NR	NR	BDI = 5.0 ± 1.8	BDI = 9.1 ± 1.8
Trung et al., 2019	N = 14 (M: 8, F: 6; age: 71.3 ± 7.3)	N = 14 (M: 11, F: 3;	10.4 ± 6.7	6.2 ± 3.0	NR	NR	33.9 ± 15.7	30.2 ± 14.5	934.0 ± 593.0	911.0 ± 699.0	All patients were PD-MCI	All patients were PD-MCI	BDI = 11.2 ± 4.3	BDI = 11.5 ± 6.6

		age: 67.3 ± 5.2)									MoCA = 24.9 ± 2.5	MoCA = 24.8 ± 2.5	BAI = 13.5 ± 5.4<br>AES = 5.1 ± 3.7	BAI = 9.1 ± 6.3<br>AES = 5.4 ± 4.1
Khedr et al., 2020	N = 18 (M: 14, F: 4; age: 65.5 ± 8.7)	N = 15 (M: 10, F: 5; age: 59.3 ± 10.3)	5.9 ± 5.4	5.5 ± 3.8	NR	NR	61.0 ± 16.4	58.0 ± 21.3	NR	NR	All patients were PD-MCI<br>CDR = 7.14 ± 3.36	All patients were PD-MCI<br>CDR = 7.1 ± 3.3	NR	NR
Lang et al., 2020	N = 21 (M: 14, F: 7; age: 68.4 ± 8.4)	N = 20 (M: 13, F: 7; age: 68.7 ± 8.3)	5.9 ± 4.8	4.8 ± 4.0	NR	NR	20.7 ± 10.2	23.5 ± 13.2	911.9 ± 522.2	930.1 ± 396.2	All patients were PD-MCI<br>MoCA = 22.9 ± 3.6	All patients were PD-MCI<br>MoCA = 22.9 ± 4.8	BDI-II = 12.3 ± 7.9<br>BAI = 13.1 ± 9.0	BDI-II = 11.0 ± 6.5<br>BAI = 12.7 ± 6.6
Li et al., 2020	N = 24 (M: 8, F: 16; age: 61.7 ± 6.9)	N = 24 (M: 8, F: 16; age: 61.5 ± 8.4)	5.5 ± 3.7	6.5 ± 5.2	1.9 ± 0.6	1.8 ± 0.6	27.8 ± 16.3	28.4 ± 15.3	435.3 ± 251.1	556.6 ± 423.0	NA	NA	HAM-D = 9.9 ± 6.3<br>HAM-A = 10.8 ± 6.6	HAM-D = 9.2 ± 6.4<br>HAM-A = 11.0 ± 7.1
Zhuang et al., 2020	N = 19 (M: 11, F: 8; age: 60.6 ± 9.2)	N = 14 (M: 7, F: 7; age: 61.6 ± 13.2)	5.9 ± 4.3	5.7 ± 3.8	2 ± 0.3	2.3 ± 0.4	27.8 ± 8.9	29.0 ± 13.2	473.9 ± 214.8	516.1 ± 210.2	MoCA = 24.4 ± 3.5	MoCA = 22.6 ± 3.1	HAM-D = 13.3 ± 6.9	HAM-D = 15.9 ± 7.1
He et al., 2021	N = 20 (M: 13, F: 7; age: 70.0 ± 6.3)	N = 15 (M: 10, F: 5; age: 74.8 ± 6.9)	2.7 ± 1.5	2.5 ± 1.1	2.7 ± 1.1	2.5 ± 1.0	36.8 ± 14.4	37.2 ± 14.9	604.4 ± 295.2	647.9 ± 323.5	All patients were PD-MCI<br>MoCA = 24.7 ± 2.9	All patients were PD-MCI<br>MoCA = 24.7 ± 3.5	BDI = 10.1 ± 8.4	BDI = 9.9 ± 8.0
Manor et al., 2021	N = 37 (M: 31, F: 6; age: 71.0 ± 8.0)	N = 36 (M: 28, F: 8; age: 69.0 ± 7.0)	10.0 ± 6.0	8.0 ± 6.0	NR	NR	40.0 ± 14.0	37.0 ± 17.0	943.0 ± 497.0	885.0 ± 596.0	Neurotrax Executive Function score = 95.0 ± 16.0	Neurotrax Executive Function score =95.0 ± 15.0	NA	NA
Aksu et al., 2022	N = 26 (M: 17, F: 9; age: 65.5 ± 7.5)		NR	NR	NR	NR	NR	NR	NR	NR	All patients were PD-MCI	All patients were PD-MCI	NA	NA
Wei et al., 2022	N = 30 (M: 18, F: 12; age: 61.7 ± 8.0)	N = 30 (M: 15, F: 15; age: 64.7 ± 9.9)	4.7 ± 1.6	5.2 ± 1.8	1 – 1.5 = 22<br>2 – 2.5 – 3 = 8	1 – 1.5 = 19<br>2 – 2.5 – 3 = 11	NR	NR	435.3 ± 242.1	382.6 ± 180.9	All PD patients had normal cognition<br>MoCA = 28.0 ± 2.4	All PD patients had normal cognition<br>MoCA = 27.3 ± 2.8	All included patients had BDI-II < 14	All included patients had BDI-II < 14
Jiang et al., 2023	N = 28 (M: 14, F: 14; age: 62.7 ± 12.9)	N = 29 (M: 12, F: 17; age: 64.3 ± 8.9)	6.8 ± 2.1	3.7 ± 1.4	1 = 1<br>1.5 = 1<br>2 = 16<br>2.5 = 5<br>3 = 4<br>4 = 1	2 = 13<br>2.5 = 14<br>3 = 2	41.7 ± 17	38.0 ± 13.0	391.5 ± 235.4	396.1 ± 340.9	MMSE = 27.7 ± 0.8<br>MoCA = 25.5 ± 1.4	MMSE = 27.7 ± 0.8<br>MoCA = 24.7 ± 1.4	All included patients met criteria for depression<br>HAM-D = 19.5 ± 7.1<br>HAM-A = 13.7 ± 6.0	All included patients met criteria for depression<br>HAM-D = 21.1 ± 6.2<br>HAM-A = 14.2 ± 4.7

Khedr et al., 2024	N = 16 (M: 7, F: 9; age: 61.8 ± 3.5)	N = 8 (M: 6, F: 2; age: 60.2 ± 1.6)	7.1 ± 3.5	5.9 ± 4.1	NR	NR	66.3 ± 23.4§	60.4 ± 33.4§	NR	NR	NA	NA	BDI = 14.9 ± 6.3	BDI = 15.4 ± 6.6
Barboza et al., 2024	N = 14 (M: 7, F: 7; age: 52.6 ± 9.3)	N = 11 (M: 4, F: 7; age: 58.4 ± 9.0)	9.3 ± 9.4	9.4 ± 5.6	NR	NR	40.0 ± 21.3	41.3 ± 11.8	893.3 ± 710.4	910.9 ± 692.3	MMSE = 26.6 ± 2.1	MMSE = 26.4 ± 2.6	HADS = 22.3 ± 8.0	HADS = 18.1 ± 5.8
Simonetta et al., 2024	N = 10 (M: 6, F: 4; age: 52.3 ± 4.2)		NR	NR	NR	NR	NR	NR	NR	NR	PD-CRS = 104.0 ± 13.53	PD-CRS = 103.6 ± 15.8	NMSS (mood/cog) = 10.1 ± 12.9	NMSS (mood/cog) = 3.2 ± 6.1
Song et al., 2024	N = 22 (M: 15, F: 7; age: 67.4 ± 7.0)	N = 22 (M: 15, F: 7; age: 70.5 ± 6.7)	6.2 ± 1.6	6.7 ± 2.0	2 – 2.5 = 15<br>3 = 7	2 – 2.5 = 14<br>3 = 8	41.7 ± 12.9	43.2 ± 10.7	706.8 ± 236.0	741.2 ± 198.0	MMSE = 26.4 ± 1.7<br>MoCA = 21.1 ± 4.2	MMSE = 25.9 ± 1.4<br>MoCA = 20.4 ± 4.0	HAM-D = 14.8 ± 6.6<br>HAM-A = 9.3 ± 4.4	HAM-D = 15.2 ± 6.2<br>HAM-A = 10.8 ± 4.9
Wu et al., 2024	N = 34 (M: 19, F: 15; age: 62.8 ± 4.4)	N = 29 (M: 14, F: 15; age: 65.9 ± 2.7)	5.2 ± 1.5	5.6 ± 1.8	2.0 ± 0.3	2.2 ± 0.4	24.0 ± 4.2	22.9 ± 5.3	459.4 ± 97.4	443.7 ± 93.8	MoCA = 24.9 ± 1.2	MoCA = 22.9 ± 1.9	NA	NA
Feng et al., 2025	N = 40 (M: 21, F: 19; age: 69.32 ± 7.61)	N = 40 (M: 22, F: 18; age: 69.42 ± 7.48)	6.23 ± 2.92	6.22 ± 3.04	2.32 ± 0.85	2.56 ± 0.79	27.01 ± 11.92	27.85 ± 10.47	NR	NR	MMSE = 23.27 ± 4.32	MMSE = 23.15 ± 4.30	HAM-D = 19.76 ± 8.79<br>HAM-A = 24.88 ± 10.34	HAM-D = 19.51 ± 8.49<br>HAM-A = 21.18 ± 7.10
Zhang et al., 2025	M1 + SMA (N = 20; M: 10, F: 10; age: 69.20 ± 5.78)<br>M1 (N = 20; M: 13, F: 7; age: 69.05 ± 6.75)<br>SMA (N = 20; M: 11, F: 9; age: 67.40 ± 7.22)	N = 19 (M: 8, F: 11; age: 71.40 ± 6.02)	M1 + SMA = 7.15 ± 3.25<br>M1 = 7.35 ± 2.88<br>SMA = 7.05 ± 2.41	7.75 ± 2.57	M1 + SMA = 2.5 (2.12, 3.0)*<br>M1 = 3 (2.5, 3.0)*<br>SMA = 2.5 (2.0, 3.0)*	3 (2.5, 3.0)	M1 + SMA = 41.30 ± 7.94<br>M1 = 40.90 ± 7.40<br>SMA = 40.85 ± 6.97	42.20 ± 7.71	M1 + SMA = 721.25 ± 274.11<br>M1 = 712.50 ± 233.72<br>SMA = 770.46 ± 258.47	740.35 ± 185.36	MoCA (M1 + SMA) = 23.55 ± 2.21<br>MoCA (M1) = 22.35 ± 3.69<br>MoCA (SMA) = 24.25 ± 2.02	MoCA = 22.00 ± 3.46	HAM-D (M1 + SMA) = 16.85 ± 2.54<br>HAM-D (M1) = 16.85 ± 3.25<br>HAM-D (SMA) = 16.80 ± 2.78<br>HAM-A (M1 + SMA) = 10.5 (8.25, 12)*<br>HAM-A (M1) = 10.5 (10, 12.75)*<br>HAM-A (SMA) = 10 (9, 13)*	HAM-D = 16.75 ± 2.55<br>HAM-A = 9 (8, 11)*

Legend to Table S1. Studies are reported in chronological order of publication. *Median, IQR. # % of patients taking ≥ 1 antidepressant medication. § Motor sections from parts II and III. **List of abbreviations.** AES = Apathy Evaluation Scale; BAI = Beck Anxiety Inventory; BDI(-II) = Beck Depression Inventory; CDR = Clinical Dementia Rating Scale; F = females; FAB = Frontal Assessment Battery; HADS-A/D = Hospital Anxiety and Depression Scale; HAM-D = Hamilton Depression Rating Scale; H-Y = Hoehn & Yahr stage; IQR = interquartile range; LEDD = levodopa equivalent daily dose; MADRS = Montgomery-Asberg Depression Rating

Scale; M = males; MDD = major depressive disorder; MMSE = Mini Mental State Examination; MoCA = Montreal Cognitive Assessment; M1 = primary motor cortex; N = number; NA = not assessed; NMS = non-motor symptoms; NMSS = Non-Motor Symptoms Scale; NR = not reported; O1 = occipital cortex; PD = Parkinson's disease; PD-CRS = Parkinson's disease Cognitive Rating Scale; SMA = supplementary motor area; UPDRS-III = Unified PD Rating Scale motor section.

Table S2. Clinical data of populations with atypical parkinsonism in the included studies

Ref.	Sample (N, sex, age)		Disease duration		H-Y		UPDRS III		LEDD (total)		Frequency/severity of cognitive NMS		Frequency/severity of neuropsychiatric NMS	
	Real	Sham	Real	Sham	Real	Sham	Real	Sham	Real	Sham	Active	Sham	Active	Sham
Elder et al., 2019*	N = 19 (M: 15, F: 4; age: 76.31 ± 8.79)	N = 17 (M: 12; F: 5; age: 73.88 ± 6.97)	NR	NR	NR	NR	36.83 ± 25.07	32.36 ± 21.93	225.63 ± 318.79	186.73 ± 264.87	MMSE = 18.16 ± 6.56 CDR = 1.24 ± 0.71	MMSE = 17.88 ± 6.06 CDR = 1.29 ± 0.56	GDS-15 = 6.81 ± 4.31	GDS-15 = 6.27 ± 2.91
Pan et al., 2022§	N = 11 (M: 5, F: 6; age: 56.84 ± 5.50)	N = 11 (M: 5, F: 6; age: 59.00 ± 6.02)	2.00 ± 1.00	1.91 ± 1.22	2.95 ± 1.19	2.86 ± 1.23	NR	NR	295.45 ± 313.41	234.09 ± 295.80	MMSE = 28.64 ± 1.86 MoCA = 23.82 ± 3.40	MMSE = 27.64 ± 1.57 MoCA = 22.91 ± 3.75	HAM-A = 16.82 ± 10.83 HAM-D = 15.27 ± 7.17	HAM-A = 14.27 ± 5.95 HAM-D = 12.45 ± 5.24
Cappiello et al., 2024#	N = 16 (M: 15, F: 1; age: 67.8 ± 6.7)	N = 9 (M: 5, F: 4; age: 67.6 ± 5.5)	5.3 ± 3.0	3.5 ± 1.5	NR	NR	NR	NR	NR	NR	MoCA = 17.1 ± 5.3	MoCA = 18.6 ± 3.3	NR	NR

Legend to Table S2. Studies are reported in chronological order of publication. *DLB + PDD patients. §MSA. #PSP. **List of abbreviations.** CDR = Clinical Dementia Rating scale; DLB = dementia with Lewy Bodies; GDS-15 = Geriatric Depression Scale (15-item version); HAM-A/D = Hamilton Anxiety/Depression Rating Scale; H-Y = Hoehn & Yahr stage; LEDD = levodopa equivalent daily dose; MMSE = Mini Mental State Examination; MoCA = Montreal Cognitive Assessment; MSA = multiple system atrophy. N = number; NR = not reported; PDD = Parkinson's disease-related dementia; PSP = Progressive Supranuclear Palsy; UPDRS-III = Unified PD Rating Scale motor section.

Table S3. Results of the sensitivity analyses on cognitive non-motor symptoms in Parkinson's disease

	Outcome and study removed for sensitivity analysis	K	N	Random-effect model results				Heterogeneity		
				MD/SMD	[95% CI]	Z	*p*	X^2	*p*	I^2 (%)
	Left DLPFC – excitatory protocols (HF rTMS, iTBS)									
End of trt	Global cognition<br>He et al., 2021	4	156	-0.24	[-0.56, 0.08]	1.48	0.14	1.80	0.62	0
	Memory<br>He et al., 2021	2	69	-0.14	[-0.61, 0.34]	0.57	0.57	0.00	0.96	0
	Language<br>He et al., 2021	2	69	-0.28	[-0.75, 0.20]	1.15	0.25	0.01	0.92	0
1 mo FU	Global cognition<br>Brys et al., 2016	3	126	0.11	[-0.24, 0.46]	0.60	0.55	0.01	0.99	0
	Bilateral M1 – excitatory protocols (HF rTMS, iTBS)									
End of trt	Global cognition<br>Khedr et al., 2020	2	73	-0.68	[-2.14, 0.78]	0.91	0.36	0.00	0.98	0
1 mo FU	Global cognition<br>Brys et al., 2016	2	79	0.83	[-1.09, 2.74]	0.84	0.40	0.25	0.62	0

Legend to Table S3. List of abbreviations. CI = confidence interval; DLPFC = dorsolateral prefrontal cortex; FU = follow-up; HF = high frequency; iTBS = intermittent theta burst stimulation; K = number of studies; MD = mean difference; mo(s) = month(s); M1 = primary motor cortex; N = number of participants; rTMS = repetitive transcranial magnetic stimulation; SMD = standardized mean difference; trt = treatment. *P* values ≤ 0.05 are reported in bold type.

Table S4. Results of the sensitivity analyses on neuropsychiatric non-motor symptoms in Parkinson's disease

	Outcome and study removed for sensitivity analysis	K	N	Random-effect model results				Heterogeneity		
				MD/SMD	[95% CI]	Z	*p*	χ^2	*p*	I^2 (%)
	Left DLPFC – excitatory protocols (HF rTMS, iTBS)									
End of trt	Depression<br>Brys et al., 2016	4	127	-0.70	[-1.23, -0.16]	2.56	**0.01**	5.72	0.13	48
	Jiang et al., 2023	4	97	-0.35	[-0.84, 0.15]	1.37	0.17	4.25	0.24	29
	Anxiety<br>Trung et al., 2019	2	84	-0.44	[-0.87, -0.00]	1.97	**0.05**	0.26	0.61	0
	Jiang et al., 2023	2	55	-0.20	[-0.73, 0.33]	0.73	0.46	0.07	0.79	0
	Apathy<br>Trung et al., 2019	2	51	1.69	[-1.95, 5.34]	0.91	0.36	0.16	0.69	0
1 mo FU	Depression<br>Brys et al., 2016	3	112	0.68	[0.17, 1.19]	2.62	**0.009**	3.33	0.19	40
	Anxiety<br>Jiang et al., 2023	2	55	0.08	[-0.45, 0.61]	0.31	0.76	0.06	0.80	0
	Bilateral M1 – excitatory protocols (HF rTMS, iTBS)									
End of trt	Depression<br>Brys et al., 2016	3	127	-0.79	[-1.15, -0.42]	4.24	**< 0.0001**	0.97	0.62	0
1 mo FU	Depression<br>Brys et al., 2016	3	127	-1.21	[-1.80, -0.62]	4.00	**< 0.0001**	4.69	0.10	57
	Bilateral M1 – excitatory protocols (HF rTMS, iTBS)									
End of trt	Anxiety<br>Brys et al., 2016	2	83	-1.05	[-1.51, -0.59]	4.45	**< 0.00001**	0.02	0.89	0
1 mo FU	Anxiety<br>Brys et al., 2016	2	83	-1.59	[-2.37, -0.80]	3.95	**< 0.0001**	2.40	0.12	58

Legend to Table S4. List of abbreviations. CI = confidence interval; DLPFC = dorsolateral prefrontal cortex; FU = follow-up; HF = high frequency; iTBS = intermittent theta burst stimulation; K = number of studies; MD = mean difference; mo(s) = month(s); M1 = primary motor cortex; N = number of participants; rTMS = repetitive transcranial magnetic stimulation; SMD = standardized mean difference; trt = treatment. *P* values ≤ 0.05 are reported in bold type.

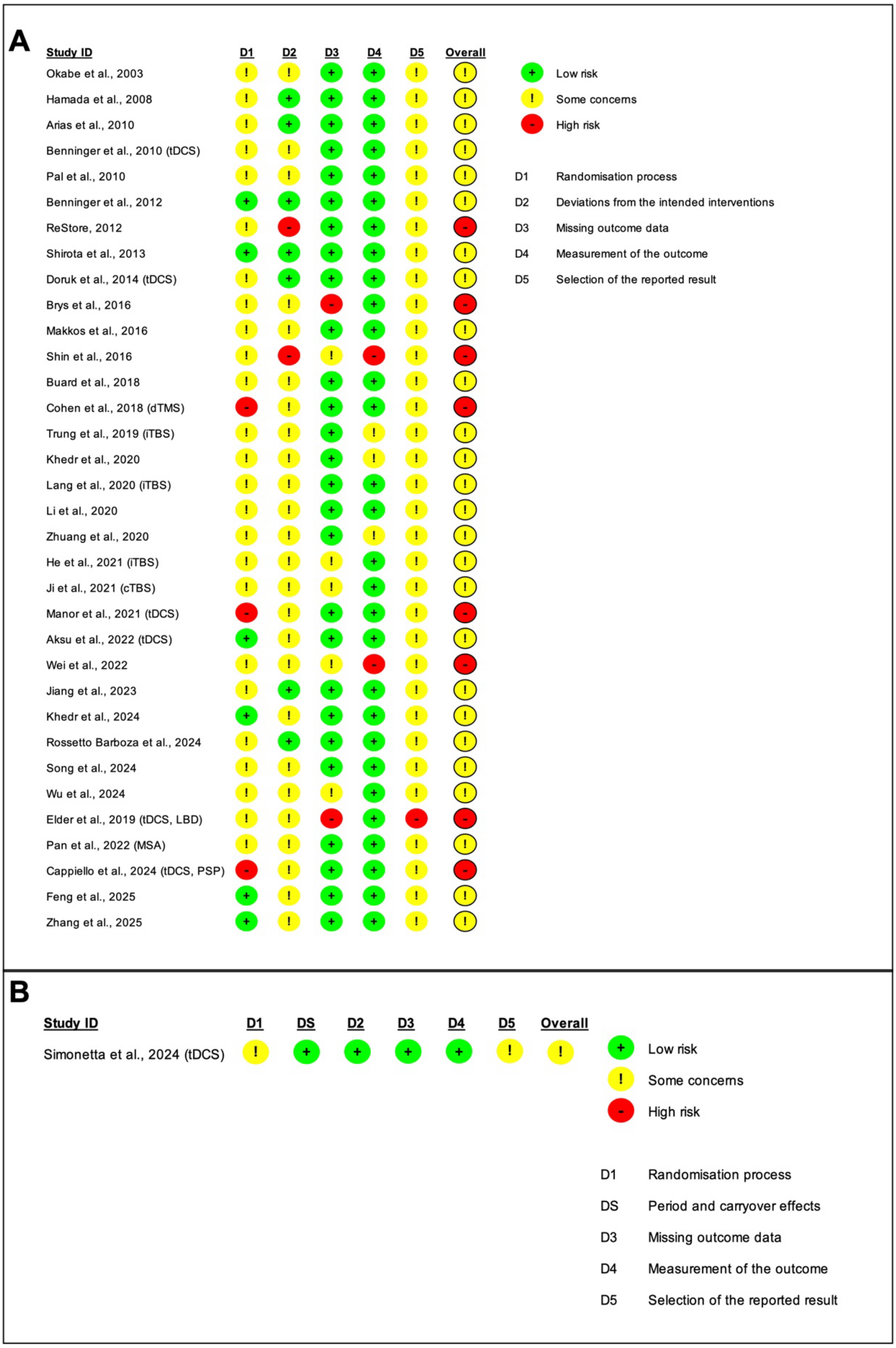

Figure S1. Risk of bias.

A - Excitatory TMS protocols (HF rTMS, iTBS) - left DLPFC - Global cognition (end of treatment)

Study or Subgroup	Active Mean	Active SD	Active Total	Sham Mean	Sham SD	Sham Total	Weight	Std. Mean Difference IV, Random, 95% CI	Year
Brys 2016	26.3	4.2	12	27.8	2.4	15	17.8%	−0.44 [−1.21, 0.33]	2016
Trung 2019	−0.33	0.49	14	−0.33	0.51	14	18.4%	0.00 [−0.74, 0.74]	2019
Lang 2020	−0.56	0.52	21	−0.56	0.68	20	21.2%	0.00 [−0.61, 0.61]	2020
He 2021	27.1	2.3	20	24.1	3.8	15	19.0%	0.97 [0.26, 1.68]	2021
Wei 2022	26.6	2.75	30	27.77	2.58	30	23.6%	−0.43 [−0.95, 0.08]	2022
**Total (95% CI)**			**97**			**94**	**100.0%**	**0.00 [−0.49, 0.49]**	

Heterogeneity: $Tau^2 = 0.20$; $Chi^2 = 11.03$, df = 4 (P = 0.03); $I^2 = 64\%$
Test for overall effect: Z = 0.02 (P = 0.99)

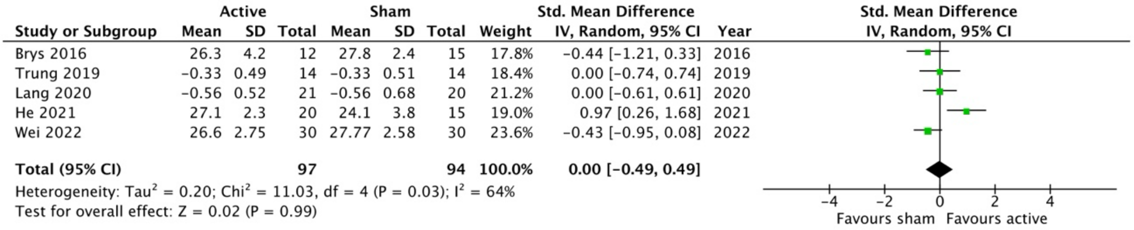

B - Excitatory TMS protocols (HF rTMS, iTBS) - left DLPFC - Global cognition (1 month FU)

Study or Subgroup	Active Mean	Active SD	Active Total	Sham Mean	Sham SD	Sham Total	Weight	Std. Mean Difference IV, Random, 95% CI
Brys 2016	26.8	2.9	12	28.7	0.9	15	19.4%	−0.90 [−1.71, −0.10]
Jang 2023	26.5	2.66	28	26.25	0.9	29	32.0%	0.13 [−0.39, 0.64]
Lang 2020	−0.41	0.63	21	−0.48	0.71	20	27.1%	0.10 [−0.51, 0.72]
Trung 2019	−0.19	0.44	14	−0.23	0.56	14	21.5%	0.08 [−0.66, 0.82]
**Total (95% CI)**			**75**			**78**	**100.0%**	**−0.09 [−0.52, 0.34]**

Heterogeneity: $Tau^2 = 0.08$; $Chi^2 = 5.13$, df = 3 (P = 0.16); $I^2 = 42\%$
Test for overall effect: Z = 0.42 (P = 0.68)

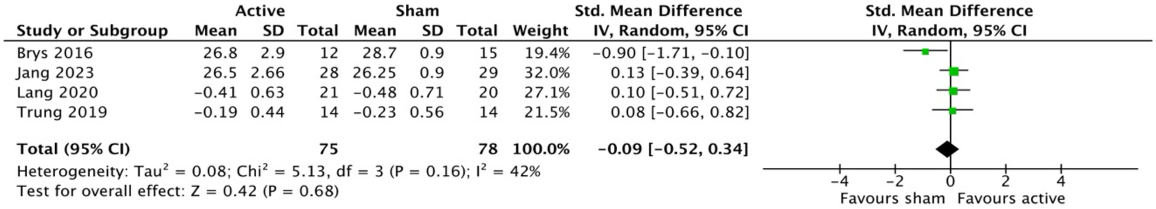

C - Excitatory TMS protocols (HF rTMS, iTBS) - left DLPFC - Global cognition (3 months FU)

Study or Subgroup	Active Mean	Active SD	Active Total	Sham Mean	Sham SD	Sham Total	Weight	Std. Mean Difference IV, Random, 95% CI
Brys 2016	26.27	3.3	12	24.9	8.4	15	45.3%	0.20 [−0.56, 0.96]
He 2021	26.7	4.1	20	24	3.1	15	54.7%	0.71 [0.02, 1.40]
**Total (95% CI)**			**32**			**30**	**100.0%**	**0.48 [−0.03, 0.99]**

Heterogeneity: $Tau^2 = 0.00$; $Chi^2 = 0.95$, df = 1 (P = 0.33); $I^2 = 0\%$
Test for overall effect: Z = 1.84 (P = 0.07)

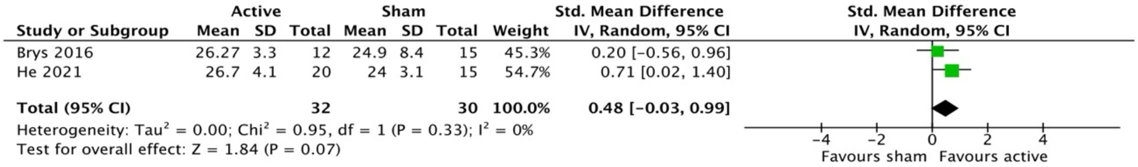

Figure S2. Forest plots of excitatory left DLPFC rTMS protocols on global cognition

A - Inhibitory TMS protocols (LF rTMS, cTBS) - right DLPFC - Global cognition (end of treatment)

Study or Subgroup	Active Mean	Active SD	Active Total	Sham Mean	Sham SD	Sham Total	Weight	Mean Difference IV, Random, 95% CI
Zhuang 2020	26.37	2.67	19	22.93	2.92	14	10.3%	3.44 [1.50, 5.38]
Wu 2024	26.25	0.9	34	23.37	1.61	29	89.7%	2.88 [2.22, 3.54]
**Total (95% CI)**			**53**			**43**	**100.0%**	**2.94 [2.31, 3.56]**

Heterogeneity: $Tau^2 = 0.00$; $Chi^2 = 0.29$, df = 1 (P = 0.59); $I^2 = 0\%$

Test for overall effect: Z = 9.22 (P < 0.00001)

Mean Difference IV, Random, 95% CI

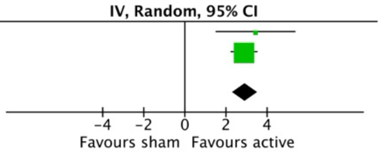

B - Inhibitory TMS protocols (LF rTMS, cTBS) - right DLPFC - Global cognition (1 month FU)

Study or Subgroup	Active Mean	Active SD	Active Total	Sham Mean	Sham SD	Sham Total	Weight	Mean Difference IV, Random, 95% CI
Zhuang 2020	26.58	3.04	19	22.29	2.87	14	10.4%	4.29 [2.26, 6.32]
Wu 2024	26.5	1.12	34	23.12	1.6	29	89.6%	3.38 [2.69, 4.07]
**Total (95% CI)**			**53**			**43**	**100.0%**	**3.47 [2.82, 4.13]**

Heterogeneity: $Tau^2 = 0.00$; $Chi^2 = 0.69$, df = 1 (P = 0.41); $I^2 = 0\%$

Test for overall effect: Z = 10.38 (P < 0.00001)

Mean Difference IV, Random, 95% CI

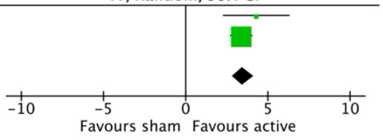

Figure S3. Forest plots of inhibitory right DLPFC protocols on global cognition

A - Excitatory TMS protocols (HF rTMS, iTBS) - bilateral M1 - Global cognition (end of treatment)

Study or Subgroup	Active Mean	Active SD	Active Total	Sham Mean	Sham SD	Sham Total	Weight	Mean Difference IV, Random, 95% CI	Year
Brys 2016	27.1	3.8	14	27.8	2.4	15	34.7%	−0.70 [−3.03, 1.63]	2016
Makkos 2016	24.33	3.16	23	25	3.18	21	40.0%	−0.67 [−2.55, 1.21]	2016
Khedr 2020	19.11	4.3	18	15.67	5.16	15	25.3%	3.44 [0.16, 6.72]	2020
**Total (95% CI)**			**55**			**51**	**100.0%**	**0.36 [−1.87, 2.59]**	

Heterogeneity: $Tau^2 = 2.33$; $Chi^2 = 5.06$, df = 2 (P = 0.08); $I^2 = 60\%$
Test for overall effect: Z = 0.32 (P = 0.75)

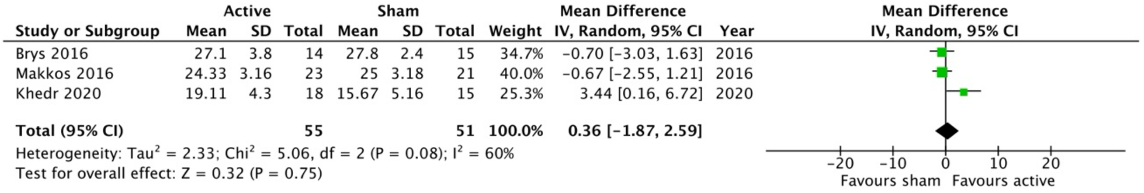

B - Excitatory TMS protocols (HF rTMS, iTBS) - bilateral M1 - Global cognition (1 month FU)

Study or Subgroup	Active Mean	Active SD	Active Total	Sham Mean	Sham SD	Sham Total	Weight	Mean Difference IV, Random, 95% CI	Year
Brys 2016	27.2	3.5	14	28.7	0.9	15	44.1%	−1.50 [−3.39, 0.39]	2016
Makkos 2016	26	1.58	23	25.53	5.57	23	34.1%	0.47 [−1.90, 2.84]	2016
Khedr 2020	17.17	4.19	18	15.67	5.19	15	21.8%	1.50 [−1.76, 4.76]	2020
**Total (95% CI)**			**55**			**53**	**100.0%**	**−0.18 [−1.92, 1.57]**	

Heterogeneity: $Tau^2 = 0.87$; $Chi^2 = 3.12$, df = 2 (P = 0.21); $I^2 = 36\%$
Test for overall effect: Z = 0.20 (P = 0.84)

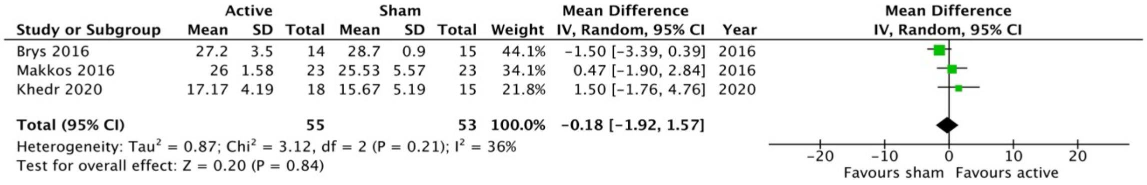

C - Excitatory TMS protocols (HF rTMS, iTBS) - bilateral M1 - Global cognition (3 months FU)

Study or Subgroup	Active Mean	Active SD	Active Total	Sham Mean	Sham SD	Sham Total	Weight	Mean Difference IV, Random, 95% CI	Year
Brys 2016	25.3	8.6	14	24.9	8.4	15	21.5%	0.40 [−5.79, 6.59]	2016
Khedr 2020	17.5	3.93	18	15.73	5.3	15	78.5%	1.77 [−1.47, 5.01]	2020
**Total (95% CI)**			**32**			**30**	**100.0%**	**1.48 [−1.39, 4.35]**	

Heterogeneity: $Tau^2 = 0.00$; $Chi^2 = 0.15$, df = 1 (P = 0.70); $I^2 = 0\%$
Test for overall effect: Z = 1.01 (P = 0.31)

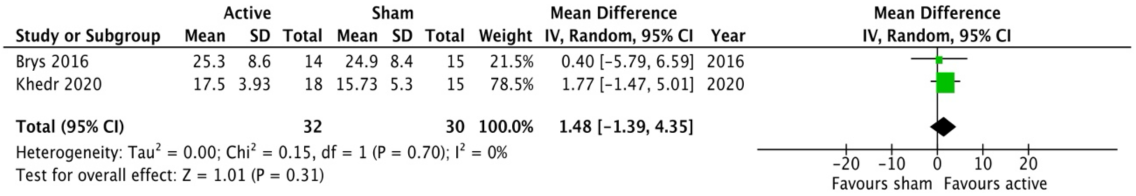

Figure S4. Forest plots of excitatory bilateral M1 rTMS protocols on global cognition

A - Excitatory TMS protocols (HF rTMS, iTBS) - left DLPFC - Attention (end of treatment)

Study or Subgroup	Active Mean	Active SD	Active Total	Sham Mean	Sham SD	Sham Total	Weight	Std. Mean Difference IV, Random, 95% CI	Year
Trung 2019	0.31	0.48	14	0.32	0.66	14	17.3%	−0.02 [−0.76, 0.72]	2019
Lang 2020	−0.39	0.61	21	−0.38	0.77	20	25.3%	−0.01 [−0.63, 0.60]	2020
He 2021	96.6	16.3	20	88.5	9.9	15	20.3%	0.57 [−0.12, 1.25]	2021
Wei 2022	68.93	18.76	30	68.47	20.18	30	37.1%	0.02 [−0.48, 0.53]	2022
**Total (95% CI)**			**85**			**79**	**100.0%**	**0.12 [−0.19, 0.43]**	

Heterogeneity: $Tau^2 = 0.00$; $Chi^2 = 2.10$, df = 3 (P = 0.55); $I^2 = 0\%$
Test for overall effect: Z = 0.75 (P = 0.46)

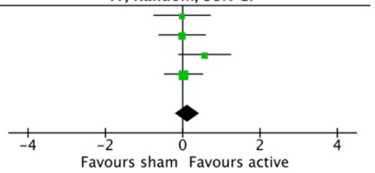

B - Excitatory TMS protocols (HF rTMS, iTBS) - left DLPFC - Attention (1 month FU)

Study or Subgroup	Active Mean	Active SD	Active Total	Sham Mean	Sham SD	Sham Total	Weight	Mean Difference IV, Random, 95% CI	Year
Trung 2019	0.33	0.51	14	0.49	0.72	14	48.7%	−0.16 [−0.62, 0.30]	2019
Lang 2020	−0.35	0.56	21	−0.33	0.87	20	51.3%	−0.02 [−0.47, 0.43]	2020
**Total (95% CI)**			**35**			**34**	**100.0%**	**−0.09 [−0.41, 0.23]**	

Heterogeneity: $Tau^2 = 0.00$; $Chi^2 = 0.18$, df = 1 (P = 0.67); $I^2 = 0\%$
Test for overall effect: Z = 0.54 (P = 0.59)

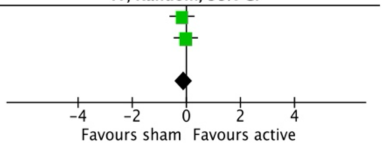

Figure S5. Forest plots of excitatory left DLPFC rTMS protocols on attention

A - Excitatory TMS protocols (HF rTMS, iTBS) - left DLPFC - Memory (end of treatment)

Study or Subgroup	Active Mean	Active SD	Active Total	Sham Mean	Sham SD	Sham Total	Weight	Std. Mean Difference IV, Random, 95% CI	Year
Trung 2019	−0.77	0.99	14	−0.65	0.93	14	29.2%	−0.12 [−0.86, 0.62]	2019
Lang 2020	−0.74	0.75	21	−0.61	0.97	20	38.0%	−0.15 [−0.76, 0.47]	2020
He 2021	102.2	21.4	20	90.6	16.3	15	32.7%	0.58 [−0.10, 1.27]	2021
**Total (95% CI)**			**55**			**49**	**100.0%**	**0.10 [−0.37, 0.57]**	

Heterogeneity: $Tau^2 = 0.05$; $Chi^2 = 2.89$, df = 2 (P = 0.24); $I^2 = 31\%$
Test for overall effect: Z = 0.42 (P = 0.68)

Std. Mean Difference
IV, Random, 95% CI

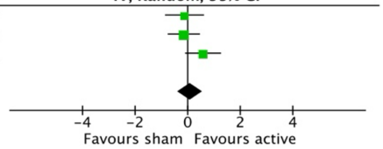

B - Excitatory TMS protocols (HF rTMS, iTBS) - left DLPFC - Memory (1 month FU)

Study or Subgroup	Active Mean	Active SD	Active Total	Sham Mean	Sham SD	Sham Total	Weight	Mean Difference IV, Random, 95% CI	Year
Trung 2019	−0.44	0.83	14	−0.59	1.05	14	36.7%	0.15 [−0.55, 0.85]	2019
Lang 2020	−0.31	0.82	21	−0.39	0.92	20	63.3%	0.08 [−0.45, 0.61]	2020
**Total (95% CI)**			**35**			**34**	**100.0%**	**0.11 [−0.32, 0.53]**	

Heterogeneity: $Tau^2 = 0.00$; $Chi^2 = 0.02$, df = 1 (P = 0.88); $I^2 = 0\%$
Test for overall effect: Z = 0.49 (P = 0.63)

Mean Difference
IV, Random, 95% CI

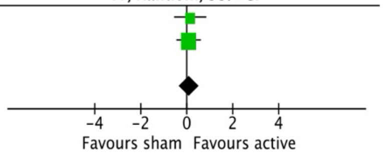

Figure S6. Forest plots of excitatory left DLPFC rTMS protocols on memory

A - Excitatory TMS protocols (HF rTMS, iTBS) - left DLPFC - Language (end of treatment)

	Active			Sham				Std. Mean Difference	
Study or Subgroup	Mean	SD	Total	Mean	SD	Total	Weight	IV, Random, 95% CI	Year
Trung 2019	−0.74	0.66	14	−0.59	0.49	14	31.0%	−0.25 [−0.99, 0.49]	2019
Lang 2020	−0.46	0.75	21	−0.21	0.89	20	36.0%	−0.30 [−0.91, 0.32]	2020
He 2021	92.5	13.3	20	84.6	8.5	15	33.0%	0.67 [−0.02, 1.36]	2021
**Total (95% CI)**			**55**			**49**	**100.0%**	**0.04 [−0.58, 0.66]**	

Heterogeneity: $Tau^2 = 0.18$; $Chi^2 = 4.95$, df = 2 (P = 0.08); $I^2 = 60\%$
Test for overall effect: Z = 0.12 (P = 0.91)

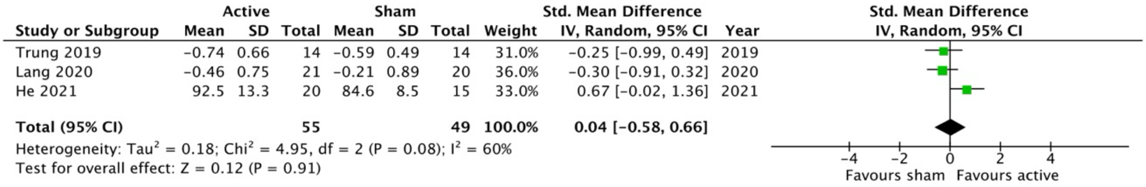

B - Excitatory TMS protocols (HF rTMS, iTBS) - left DLPFC - Language (1 month FU)

	Active			Sham				Mean Difference	
Study or Subgroup	Mean	SD	Total	Mean	SD	Total	Weight	IV, Random, 95% CI	Year
Trung 2019	−0.53	0.61	14	−0.58	0.64	14	56.0%	0.05 [−0.41, 0.51]	2019
Lang 2020	−0.38	0.79	21	−0.29	0.91	20	44.0%	−0.09 [−0.61, 0.43]	2020
**Total (95% CI)**			**35**			**34**	**100.0%**	**-0.01 [−0.36, 0.34]**	

Heterogeneity: $Tau^2 = 0.00$; $Chi^2 = 0.15$, df = 1 (P = 0.69); $I^2 = 0\%$
Test for overall effect: Z = 0.07 (P = 0.95)

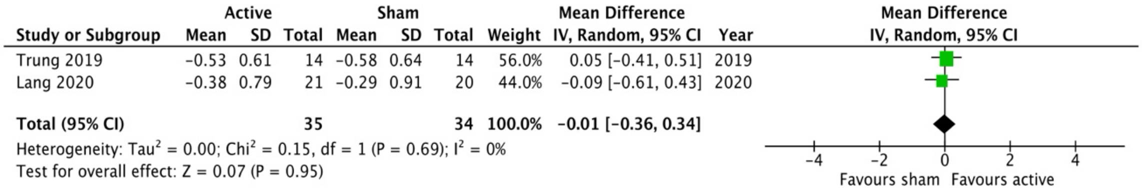

Figure S7. Forest plots of excitatory left DLPFC rTMS protocols on language

A - Excitatory TMS protocols (HF rTMS, iTBS) - left DLPFC - Visuospatial function (end of treatment)

	Active			Sham				Std. Mean Difference	
Study or Subgroup	Mean	SD	Total	Mean	SD	Total	Weight	IV, Random, 95% CI	Year
Trung 2019	−0.08	0.45	14	−0.23	0.6	14	27.3%	0.27 [−0.47, 1.02]	2019
Lang 2020	−0.75	0.78	21	−1.1	0.86	20	39.4%	0.42 [−0.20, 1.04]	2020
He 2021	95.7	18.1	20	90.1	20	15	33.4%	0.29 [−0.38, 0.96]	2021
**Total (95% CI)**			**55**			**49**	**100.0%**	**0.34 [−0.05, 0.73]**	

Heterogeneity: $Tau^2 = 0.00$; $Chi^2 = 0.11$, df = 2 (P = 0.95); $I^2 = 0\%$
Test for overall effect: Z = 1.69 (P = 0.09)

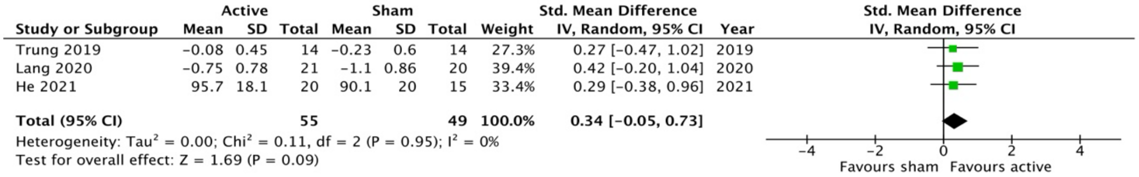

B - Excitatory TMS protocols (HF rTMS, iTBS) - left DLPFC - Visuospatial function (1 month FU)

	Active			Sham				Mean Difference	
Study or Subgroup	Mean	SD	Total	Mean	SD	Total	Weight	IV, Random, 95% CI	Year
Trung 2019	−0.04	0.58	14	−0.15	0.52	14	63.8%	0.11 [−0.30, 0.52]	2019
Lang 2020	−0.59	0.93	21	−0.83	0.84	20	36.2%	0.24 [−0.30, 0.78]	2020
**Total (95% CI)**			**35**			**34**	**100.0%**	**0.16 [−0.17, 0.48]**	

Heterogeneity: $Tau^2 = 0.00$; $Chi^2 = 0.14$, df = 1 (P = 0.71); $I^2 = 0\%$
Test for overall effect: Z = 0.94 (P = 0.35)

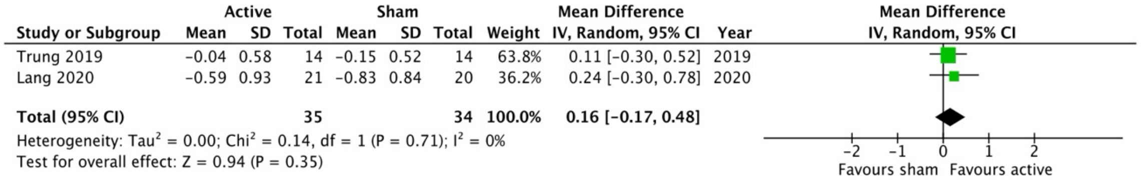

Figure S8. Forest plots of excitatory left DLPFC rTMS protocols on visuospatial function

A - Excitatory TMS protocols (HF rTMS, iTBS) - left DLPFC - Executive function (end of treatment)

	Active			Sham				Std. Mean Difference	
Study or Subgroup	Mean	SD	Total	Mean	SD	Total	Weight	IV, Random, 95% CI	Year
Trung 2019	−0.36	0.67	14	−0.52	0.96	14	21.7%	0.19 [−0.56, 0.93]	2019
Lang 2020	−0.47	0.65	21	−0.54	0.86	20	31.8%	0.09 [−0.52, 0.70]	2020
Wei 2022	8.47	5.57	30	9.37	5.79	30	46.5%	−0.16 [−0.66, 0.35]	2022
**Total (95% CI)**			**65**			**64**	**100.0%**	**−0.00 [−0.35, 0.34]**	

Heterogeneity: $Tau^2 = 0.00$; $Chi^2 = 0.69$, df = 2 (P = 0.71); $I^2 = 0\%$
Test for overall effect: Z = 0.02 (P = 0.99)

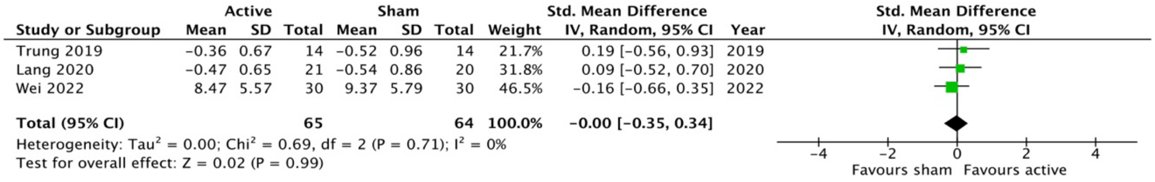

B - Excitatory TMS protocols (HF rTMS, iTBS) - left DLPFC - Executive function (1 month FU)

	Active			Sham				Mean Difference	
Study or Subgroup	Mean	SD	Total	Mean	SD	Total	Weight	IV, Random, 95% CI	Year
Trung 2019	−0.25	0.57	14	−0.32	1.02	14	44.0%	0.07 [−0.54, 0.68]	2019
Lang 2020	−0.45	0.85	21	−0.56	0.92	20	56.0%	0.11 [−0.43, 0.65]	2020
**Total (95% CI)**			**35**			**34**	**100.0%**	**0.09 [−0.31, 0.50]**	

Heterogeneity: $Tau^2 = 0.00$; $Chi^2 = 0.01$, df = 1 (P = 0.92); $I^2 = 0\%$
Test for overall effect: Z = 0.45 (P = 0.66)

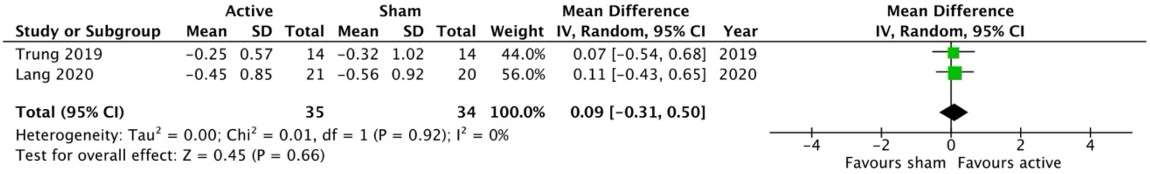

Figure S9. Forest plots of excitatory left DLPFC rTMS protocols on executive function

A - Excitatory TMS protocols (HF rTMS, iTBS) - left DLPFC - Depression (end of treatment)

Study or Subgroup	Active Mean	Active SD	Active Total	Sham Mean	Sham SD	Sham Total	Weight	Std. Mean Difference IV, Random, 95% CI	Year
ReStore 2012 (NCT00955032)	7.9	4.8	16	8.5	5.4	8	18.3%	−0.12 [−0.97, 0.73]	
Brys 2016	9.4	5.9	12	9.3	5.9	15	20.5%	0.02 [−0.74, 0.78]	2016
Shin 2016	9.7	4.3	10	17.6	7.4	8	14.6%	−1.28 [−2.33, −0.24]	2016
Trung 2019	8.42	5.47	14	10.42	6.61	14	20.8%	−0.32 [−1.07, 0.43]	2019
Jang 2023	12.5	5.5	28	18.5	5.5	29	25.9%	−1.08 [−1.63, −0.52]	2023
**Total (95% CI)**			**80**			**74**	**100.0%**	**−0.55 [−1.06, −0.04]**	

Heterogeneity: $Tau^2 = 0.18$; $Chi^2 = 8.76$, df = 4 (P = 0.07); $I^2 = 54\%$
Test for overall effect: Z = 2.12 (P = 0.03)

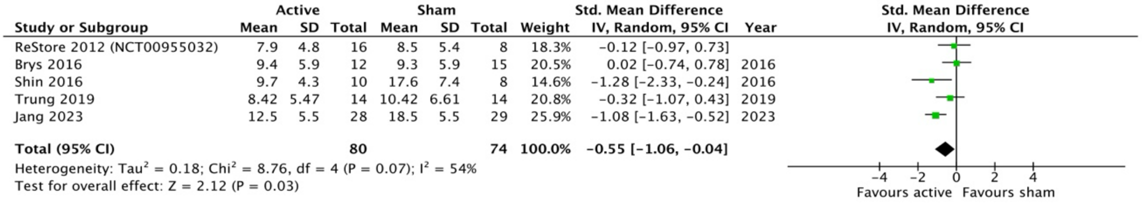

B - Excitatory TMS protocols (HF rTMS, iTBS) - left DLPFC - Depression (1 month FU)

Study or Subgroup	Active Mean	Active SD	Active Total	Sham Mean	Sham SD	Sham Total	Weight	Std. Mean Difference IV, Random, 95% CI	Year
Brys 2016	12.4	8.1	12	8	3.7	15	24.8%	0.71 [−0.08, 1.49]	2016
Shin 2016	12.3	3.2	10	18.2	7.9	8	21.5%	−0.98 [−1.97, 0.02]	2016
Trung 2019	11.07	6.15	14	9.85	7.6	14	25.4%	0.17 [−0.57, 0.91]	2019
Jang 2023	17.4	7.5	28	10.7	5.1	29	28.3%	1.03 [0.48, 1.59]	2023
**Total (95% CI)**			**64**			**66**	**100.0%**	**0.30 [−0.48, 1.08]**	

Heterogeneity: $Tau^2 = 0.48$; $Chi^2 = 12.99$, df = 3 (P = 0.005); $I^2 = 77\%$
Test for overall effect: Z = 0.76 (P = 0.45)

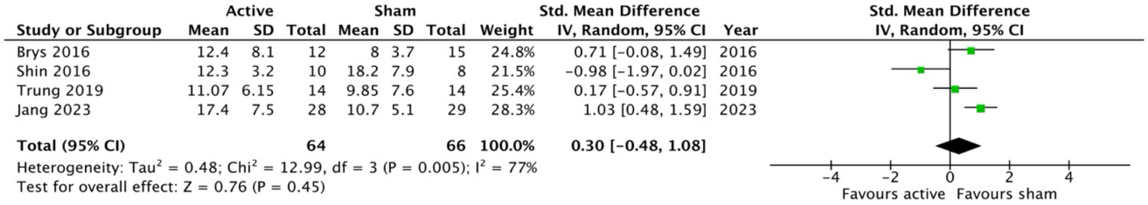

Figure S10. Forest plots of excitatory left DLPFC rTMS protocols on depression

A - Inhibitory TMS protocols (LF rTMS, cTBS) - right DLPFC - Depression (1 month FU)

	Active			Sham				Mean Difference
Study or Subgroup	Mean	SD	Total	Mean	SD	Total	Weight	IV, Random, 95% CI
Zhuang 2020	10.32	5.33	19	15.57	7.23	14	31.6%	−5.25 [−9.73, −0.77]
Feng 2025	19.08	7.11	40	23.98	6.78	40	68.4%	−4.90 [−7.94, −1.86]
**Total (95% CI)**			**59**			**54**	**100.0%**	**−5.01 [−7.53, −2.49]**

Heterogeneity: $Tau^2 = 0.00$; $Chi^2 = 0.02$, $df = 1$ ($P = 0.90$); $I^2 = 0\%$

Test for overall effect: $Z = 3.90$ ($P < 0.0001$)

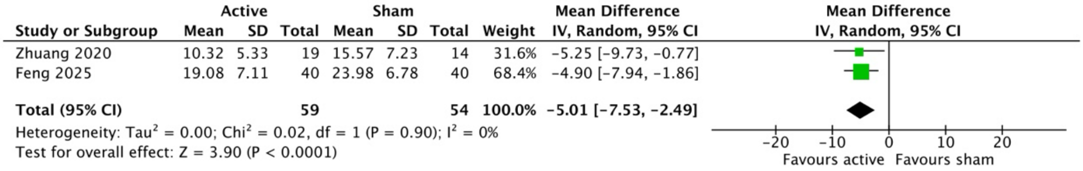

Figure S11. Forest plot of inhibitory right DLPFC rTMS protocols on depression

A - Excitatory TMS protocols (HF rTMS, iTBS) - Bilateral M1 - Depression (end of treatment)

	Active			Sham				Std. Mean Difference	
Study or Subgroup	Mean	SD	Total	Mean	SD	Total	Weight	IV, Random, 95% CI	Year
Brys 2016	11.2	6.3	14	9.3	5.9	15	22.8%	0.30 [−0.43, 1.04]	2016
Makkos 2016	7.67	7.11	23	12.33	7.16	21	26.4%	−0.64 [−1.25, −0.03]	2016
Song 2024	11.05	6.42	22	15.55	6.21	22	26.4%	−0.70 [−1.31, −0.09]	2024
Zhang 2025	13.85	2.7	20	16.95	2.98	19	24.4%	−1.07 [−1.75, −0.39]	2025
**Total (95% CI)**			**79**			**77**	**100.0%**	**−0.55 [−1.07, −0.02]**	

Heterogeneity: $Tau^2 = 0.18$; $Chi^2 = 7.77$, df = 3 (P = 0.05); $I^2 = 61\%$
Test for overall effect: Z = 2.03 (P = 0.04)

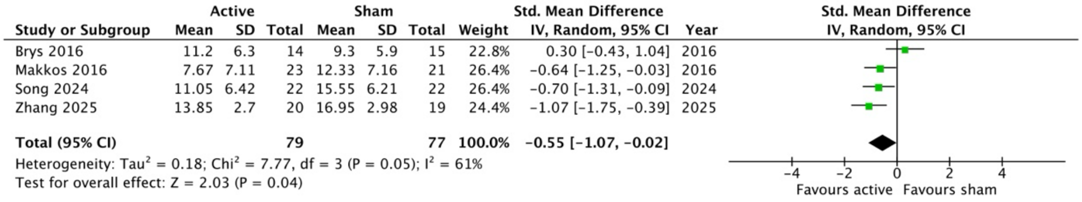

B - Excitatory TMS protocols (HF rTMS, iTBS) - Bilateral M1 - Depression (1 month FU)

	Active			Sham				Std. Mean Difference	
Study or Subgroup	Mean	SD	Total	Mean	SD	Total	Weight	IV, Random, 95% CI	Year
Makkos 2016	8	5.53	23	13.67	6.36	21	25.7%	−0.94 [−1.56, −0.31]	2016
Brys 2016	10.1	5.4	14	8	3.7	15	24.4%	0.44 [−0.29, 1.18]	2016
Song 2024	9.5	6.67	22	15.45	6.19	22	25.8%	−0.91 [−1.53, −0.28]	2024
Zhang 2025	12.35	1.69	20	16.85	2.83	19	24.1%	−1.90 [−2.67, −1.13]	2025
**Total (95% CI)**			**79**			**77**	**100.0%**	**−0.82 [−1.69, 0.04]**	

Heterogeneity: $Tau^2 = 0.65$; $Chi^2 = 19.07$, df = 3 (P = 0.0003); $I^2 = 84\%$
Test for overall effect: Z = 1.87 (P = 0.06)

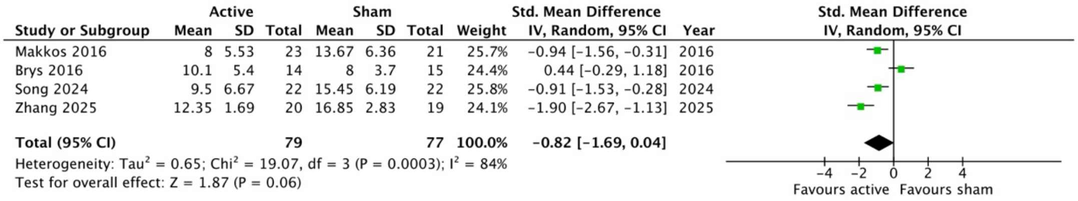

Figure S12. Forest plots of excitatory bilateral M1 rTMS protocols on depression

A - Excitatory TMS protocols (HF rTMS, iTBS) - left DLPFC - Anxiety (end of treatment)

Study or Subgroup	Active Mean	Active SD	Active Total	Sham Mean	Sham SD	Sham Total	Weight	Std. Mean Difference IV, Random, 95% CI	Year
Brys 2016	27.6	17.4	12	32.4	16.8	15	24.1%	−0.27 [−1.04, 0.49]	2016
Trung 2019	12.28	5.35	14	13.5	11.73	14	25.5%	−0.13 [−0.87, 0.61]	2019
Jang 2023	9.6	5.4	28	12.1	4.1	29	50.3%	−0.52 [−1.04, 0.01]	2023
**Total (95% CI)**			**54**			**58**	**100.0%**	**−0.36 [−0.73, 0.02]**	

Heterogeneity: $Tau^2 = 0.00$; $Chi^2 = 0.75$, df = 2 (P = 0.69); $I^2 = 0\%$
Test for overall effect: Z = 1.87 (P = 0.06)

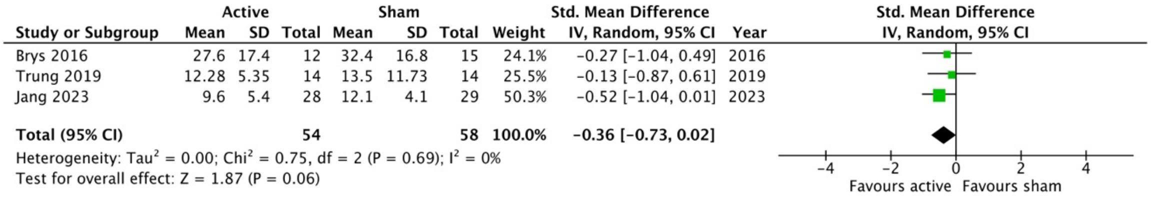

B - Excitatory TMS protocols (HF rTMS, iTBS) - left DLPFC - Anxiety (1 month FU)

Study or Subgroup	Active Mean	Active SD	Active Total	Sham Mean	Sham SD	Sham Total	Weight	Std. Mean Difference IV, Random, 95% CI	Year
Brys 2016	30.8	17.7	12	28.2	15.3	15	29.2%	0.15 [−0.61, 0.91]	2016
Trung 2019	10.85	5.74	14	10.71	10.77	14	30.0%	0.02 [−0.73, 0.76]	2019
Jang 2023	8.7	3.6	28	11.9	5.5	29	40.8%	−0.68 [−1.21, −0.14]	2023
**Total (95% CI)**			**54**			**58**	**100.0%**	**−0.23 [−0.77, 0.32]**	

Heterogeneity: $Tau^2 = 0.12$; $Chi^2 = 3.97$, df = 2 (P = 0.14); $I^2 = 50\%$
Test for overall effect: Z = 0.81 (P = 0.42)

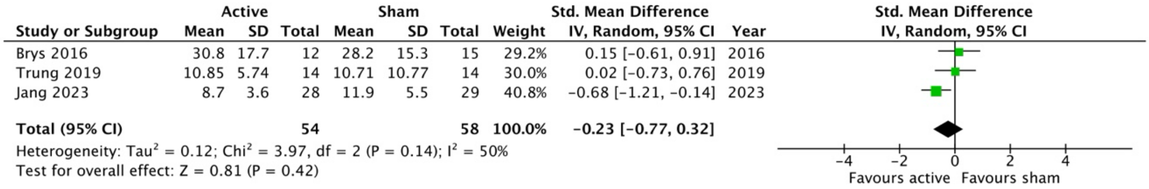

Figure S13. Forest plots of excitatory left DLPFC rTMS protocols on anxiety

A - Excitatory TMS protocols (HF rTMS, iTBS) - Bilateral M1 - Anxiety (end of treatment)

	Active			Sham				Std. Mean Difference	
**Study or Subgroup**	**Mean**	**SD**	**Total**	**Mean**	**SD**	**Total**	**Weight**	**IV, Random, 95% CI**	**Year**
Brys 2016	31	13.8	14	32.4	16.8	15	31.3%	−0.09 [−0.82, 0.64]	2016
Song 2024	6.18	4.22	22	10.73	4.55	22	35.3%	−1.02 [−1.65, −0.39]	2024
Zhang 2025	8.55	2.67	20	10.95	1.47	19	33.4%	−1.08 [−1.76, −0.41]	2025
**Total (95% CI)**			**56**			**56**	**100.0%**	**−0.75 [−1.35, −0.14]**	

Heterogeneity: $Tau^2 = 0.17$; $Chi^2 = 4.78$, df = 2 (P = 0.09); $I^2 = 58\%$
Test for overall effect: Z = 2.42 (P = 0.02)

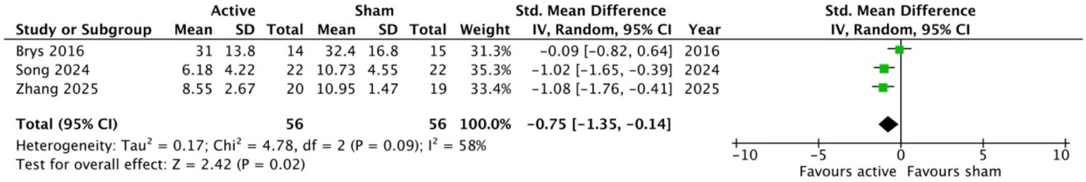

B - Excitatory TMS protocols (HF rTMS, iTBS) - Bilateral M1 - Anxiety (1 month FU)

	Active			Sham				Std. Mean Difference	
**Study or Subgroup**	**Mean**	**SD**	**Total**	**Mean**	**SD**	**Total**	**Weight**	**IV, Random, 95% CI**	**Year**
Brys 2016	30.8	12.9	14	28.2	15.3	15	33.3%	0.18 [−0.55, 0.91]	2016
Song 2024	5.5	3.7	22	10.82	4.82	22	34.1%	−1.22 [−1.86, −0.57]	2024
Zhang 2025	7.15	2.23	20	11	1.38	19	32.6%	−2.02 [−2.81, −1.24]	2025
**Total (95% CI)**			**56**			**56**	**100.0%**	**−1.02 [−2.22, 0.19]**	

Heterogeneity: $Tau^2 = 1.00$; $Chi^2 = 16.91$, df = 2 (P = 0.0002); $I^2 = 88\%$
Test for overall effect: Z = 1.65 (P = 0.10)

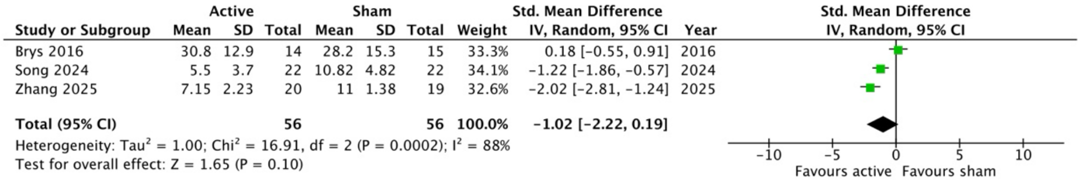

Figure S14. Forest plots of excitatory bilateral M1 rTMS protocols on anxiety

A - Excitatory TMS protocols (HF rTMS, iTBS) - left DLPFC - Apathy (end of treatment)

Study or Subgroup	Active Mean	Active SD	Active Total	Sham Mean	Sham SD	Sham Total	Weight	Mean Difference IV, Random, 95% CI	Year
ReStore 2012 (NCT00955032)	17.9	7	16	16.8	4.6	8	27.5%	1.10 [−3.58, 5.78]	
Brys 2016	18.1	8.8	12	15.5	5.9	15	17.9%	2.60 [−3.21, 8.41]	2016
Trung 2019	5.64	4.9	14	5.35	4.04	14	54.6%	0.29 [−3.04, 3.62]	2019
**Total (95% CI)**			**42**			**37**	**100.0%**	**0.93 [−1.53, 3.38]**	

Heterogeneity: $Tau^2 = 0.00$; $Chi^2 = 0.47$, df = 2 (P = 0.79); $I^2 = 0\%$

Test for overall effect: Z = 0.74 (P = 0.46)

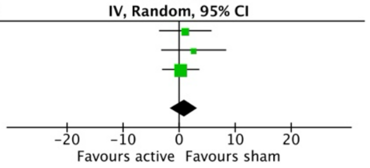

B - Excitatory TMS protocols (HF rTMS, iTBS) - left DLPFC - Apathy (1 month FU)

Study or Subgroup	Active Mean	Active SD	Active Total	Sham Mean	Sham SD	Sham Total	Weight	Mean Difference IV, Random, 95% CI	Year
Brys 2016	19	9.7	12	15	5.3	15	40.1%	4.00 [−2.11, 10.11]	2016
Trung 2019	5.5	4.75	14	6.6	6.45	14	59.9%	−1.10 [−5.30, 3.10]	2019
**Total (95% CI)**			**26**			**29**	**100.0%**	**0.95 [−3.95, 5.85]**	

Heterogeneity: $Tau^2 = 5.86$; $Chi^2 = 1.82$, df = 1 (P = 0.18); $I^2 = 45\%$

Test for overall effect: Z = 0.38 (P = 0.70)

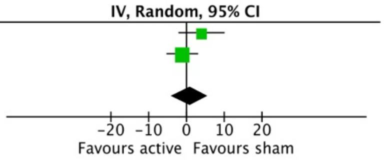

Figure S15. Forest plots of excitatory left DLPFC rTMS protocols on apathy

Supplementary figure legends

Figure S1. Risk of bias. Traffic light plots showing the assessment of the risk of bias for included randomized-controlled trials with parallel (panel A) and cross-over (panel B) designs.

Figure S2. Forest plots of excitatory left DLPFC rTMS protocols on global cognition. Here are reported global cognition outcome measures to real/active vs sham excitatory repetitive transcranial stimulation (rTMS) of the left dorsolateral prefrontal cortex (DLPFC) at different time points (i.e., end of treatment, panel A; 1-month follow-up, panel B; 3-month follow-up, panel C). Standardized Mean Difference (SMD) represents Hedges's g effect size. The size of the square indicates the weight of the study. The horizontal line represents the 95% confidence interval. The diamond represents the pooled effect size. Positive effect sizes favour real/active rTMS vs sham.

Figure S3. Forest plots of inhibitory right DLPFC rTMS protocols on global cognition. Here are reported global cognition outcome measures to real/active vs sham inhibitory repetitive transcranial stimulation (rTMS) of the right dorsolateral prefrontal cortex (DLPFC) at different time points (i.e., post-treatment, panel A; 3-month follow-up, panel B). The size of the square indicates the weight of the study. The horizontal line represents the 95% confidence interval. The diamond represents the pooled effect size. Positive effect sizes favour real/active rTMS over sham.

Figure S4. Forest plots of excitatory bilateral M1 rTMS protocols on global cognition. Here are reported global cognition outcome measures to real/active vs sham excitatory repetitive transcranial stimulation (rTMS) of the bilateral primary motor cortex (M1) at different time points (i.e., post-treatment, panel A; 1-month follow-up, panel B; 3-month follow-up, panel C). The size of the square indicates the weight of the study. The horizontal line represents the 95% confidence interval. The diamond represents the pooled effect size. Positive effect sizes favour real/active rTMS over sham.

Figure S5. Forest plots of excitatory left DLPFC rTMS protocols on attention. Here are reported attention outcome measures to real/active vs sham excitatory repetitive transcranial stimulation (rTMS) of the left dorsolateral prefrontal cortex (DLPFC) at different time points (i.e., end of treatment, panel A; 1-month follow-up, panel B). Standardized Mean Difference (SMD) represents Hedges's g effect size. The size of the square indicates the weight of the study. The horizontal line represents the 95% confidence interval. The diamond represents the pooled effect size. Positive effect sizes favour real/active rTMS over sham.

Figure S6. Forest plots of excitatory left DLPFC rTMS protocols on memory. Here are reported memory outcome measures to real/active vs sham excitatory repetitive transcranial stimulation (rTMS) of the left dorsolateral prefrontal cortex (DLPFC) at different time points (i.e., end of treatment, panel A; 1-month follow-up, panel B). Standardized Mean Difference (SMD) represents Hedges's g effect size. The size of the square indicates the weight of the study. The horizontal line represents the 95% confidence interval. The diamond represents the pooled effect size. Positive effect sizes favour real/active rTMS over sham.

Figure S7. Forest plots of excitatory left DLPFC rTMS protocols on language. Here are reported language outcome measures to real/active vs sham excitatory repetitive transcranial stimulation (rTMS) of the left dorsolateral prefrontal cortex (DLPFC) at different time points (i.e., end of treatment, panel A; 1-month follow-up, panel B). Standardized Mean Difference (SMD) represents Hedges's g effect size. The size of the square indicates the weight of the study. The horizontal line represents the 95% confidence interval. The diamond represents the pooled effect size. Positive effect sizes favour real/active rTMS over sham.

Figure S8. Forest plots of excitatory left DLPFC rTMS protocols on visuospatial function. Here are reported visuospatial function outcome measures to real/active vs sham excitatory repetitive transcranial stimulation (rTMS) of the left dorsolateral prefrontal cortex (DLPFC) at different time points (i.e., end of treatment, panel A; 1-month follow-up, panel B). Standardized Mean Difference (SMD) represents Hedges's g effect size. The size of the square indicates the weight of the study. The horizontal line represents the 95% confidence interval. The diamond represents the pooled effect size. Positive effect sizes favour real/active rTMS over sham.

Figure S9. Forest plots of excitatory left DLPFC rTMS protocols on executive function. Here are reported executive function outcome measures to real/active vs sham excitatory repetitive transcranial stimulation (rTMS) of the left dorsolateral prefrontal cortex (DLPFC) at different time points (i.e., end of treatment, panel A; 1-month follow-up, panel B). Standardized Mean Difference (SMD) represents Hedges's g effect size. The size of the square indicates the weight of the study. The horizontal line represents the 95% confidence interval. The diamond represents the pooled effect size. Positive effect sizes favour real/active rTMS over sham.

Figure S10. Forest plots of excitatory left DLPFC rTMS protocols on depression. Here are reported depression outcome measures to real/active vs sham excitatory repetitive transcranial stimulation (rTMS) of the left dorsolateral prefrontal cortex (DLPFC) at different time points (i.e., end of treatment, panel A; 1-month follow-up, panel B). Standardized Mean Difference (SMD) represents Hedges's g effect size. The size of the square indicates the weight of the study. The horizontal line represents the 95% confidence interval. The diamond represents the pooled effect size. Negative effect sizes favour real/active rTMS over sham.

Figure S11. Forest plots of inhibitory right DLPFC rTMS protocols on depression. Here are reported depression outcome measures to real/active vs sham inhibitory repetitive transcranial stimulation (rTMS) of the right dorsolateral prefrontal cortex (DLPFC) at 1-month follow-up. The size of the square indicates the weight of the study. The horizontal line represents the 95% confidence interval. The diamond represents the pooled effect size. Negative effect sizes favour real/active rTMS over sham.

Figure S12. Forest plots of excitatory bilateral M1 rTMS protocols on depression. Here are reported depression outcome measures to real/active vs sham excitatory repetitive transcranial stimulation (rTMS) of the bilateral primary motor cortex (M1) at different time points (i.e., end of treatment, panel A; 1-month follow-up, panel B). Standardized Mean Difference (SMD) represents Hedges's g effect size. The size of the square indicates the weight of the study. The horizontal line represents the 95% confidence interval. The diamond represents the pooled effect size. Negative effect sizes favour real/active rTMS over sham.

Figure S13. Forest plots of excitatory left DLPFC rTMS protocols on anxiety. Here are reported anxiety outcome measures to real/active vs sham excitatory repetitive transcranial stimulation (rTMS) of the left dorsolateral prefrontal cortex (DLPFC) at different time points (i.e., end of treatment, panel A; 1-month follow-up, panel B). Standardized Mean Difference (SMD) represents Hedges's g effect size. The size of the square indicates the weight of the study. The horizontal line represents the 95% confidence interval. The diamond represents the pooled effect size. Negative effect sizes favour real/active rTMS over sham.

Figure S14. Forest plots of excitatory bilateral M1 rTMS protocols on anxiety. Here are reported anxiety outcome measures to real/active vs sham excitatory repetitive transcranial stimulation (rTMS) of the bilateral primary motor cortex (M1) at different time points (i.e., end of treatment, panel A; 1-month follow-up, panel B). Standardized Mean Difference (SMD) represents Hedges's g effect size. The size of the square indicates the weight of the study. The horizontal line represents the 95% confidence interval. The diamond represents the pooled effect size. Negative effect sizes favour real/active rTMS over sham.

Figure S15. Forest plots of excitatory left DLPFC rTMS protocols on apathy. Here are reported apathy outcome measures to real/active vs sham excitatory repetitive transcranial stimulation (rTMS) of the left dorsolateral prefrontal cortex (DLPFC) at different time points (i.e., end of treatment, panel A; 1-month follow-up, panel B). The size of the square indicates the weight of the study. The horizontal line represents the 95% confidence interval. The diamond represents the pooled effect size. Negative effect sizes favour real/active rTMS over sham
